# Characterization of the Two-Domain Peptide Binding Mechanism of the Human CGRP Receptor for CGRP and the Ultrahigh Affinity ssCGRP Variant

**DOI:** 10.1021/acs.biochem.4c00812

**Published:** 2025-04-02

**Authors:** Katie M. Babin, Ceren Kilinc, Sandra E. Gostynska, Alex Dickson, Augen A. Pioszak

**Affiliations:** † Department of Biochemistry and Physiology, University of Oklahoma Health Sciences Center, Oklahoma City, Oklahoma 73104, United States; ‡ Department of Biochemistry and Molecular Biology, Michigan State University, East Lansing, Michigan 48824, United States; § Department of Computational Mathematics, Science and Engineering, Michigan State University, East Lansing, Michigan 48824, United States

## Abstract

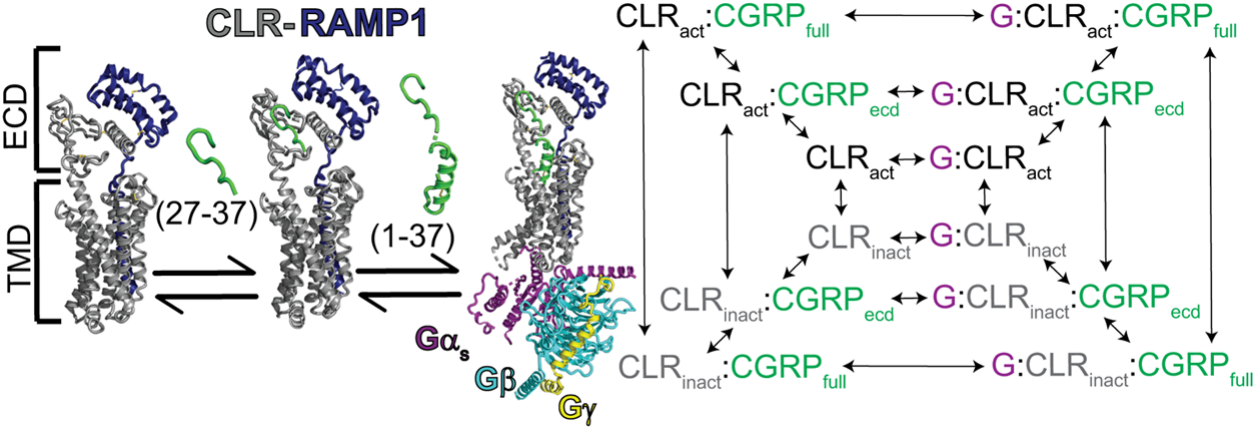

Calcitonin gene-related peptide (CGRP) is a 37-amino acid neuropeptide that functions in pain signaling and neuroimmune communication. The CGRP receptor, CGRPR, is a class B GPCR that is a drug target for migraine headache and other disorders. Here, we used nanoBRET receptor binding and cAMP biosensor signaling assays and theoretical modeling to characterize the CGRPR “two-domain” peptide binding mechanism. Single-site extracellular domain (ECD)-binding and two-site ECD/transmembrane domain (TMD)-binding peptides were examined for CGRP and a high-affinity variant “ssCGRP” with modifications in the C-terminal region. Wildtype and ssCGRP(27-37) bound the ECD with affinities of 1 μM and 0.5 nM, and residence times of 5 s and 8 min, respectively. The (8-37) antagonist fragments had affinities of 100 nM for wildtype and 0.5 nM for ss and exhibited behavior consistent with two-site ECD/TMD binding. ssCGRP(8-37) had a residence time of 76 min. CGRP(1-37) agonist had 25-fold higher affinity for the G protein-coupled state of the CGRPR (*K_i_* = 3 nM) than the uncoupled state (*K_i_* = 74 nM), and elicited short-duration cAMP signaling. In contrast, ssCGRP(1-37) had similar strong affinities for both receptor states (*K_i_* = 0.2 to 0.25 nM), and induced long-duration signaling. An equilibrium reaction network mathematical model of CGRPR activation that includes peptide and G protein binding was developed. This captured wildtype CGRP binding experiments well, but the ssCGRP binding properties were not fully reproduced, suggesting that it may exhibit a distinct binding mechanism. Together, these results advance our quantitative understanding of the CGRPR two-domain mechanism and support the ss variants as potential long-acting therapeutics.

## Introduction

Calcitonin gene-related peptide (CGRP) is a 37-amino acid neuropeptide that is released from nerve terminals and acts on diverse target cells by activating the CGRP receptor (CGRPR).^1,2^ Originally described as a vasodilator, CGRP has since been shown to have many functions. It is perhaps best known for its role in pain signaling and migraine headache pathogenesis.^3,4^ Several CGRP blocker drugs including small molecule receptor antagonists and monoclonal antibodies targeting either the receptor or ligand are in clinical use for migraine.^5^ CGRP has cardioprotective effects and CGRPR agonists may have potential as therapeutics for cardiovascular disorders.^6,7^ There has been intense recent interest in the roles of CGRP in communication between the nervous and immune systems.^8^ Here, CGRP has functions relevant to viral transmission, bacterial infections, cancer, and wound healing.^9−13^ Depending on the situation, either antagonists or agonists of the CGRPR may have therapeutic value for modulating the immune system. For drug development it is critical to understand the receptor ligand binding and activation mechanisms and the properties of potential antagonist and agonist drugs at the receptor.

The CGRPR is a heterodimer of the calcitonin receptor-like receptor (CLR) and receptor activity-modifying protein 1 (RAMP1).^14^ CLR is a class B G protein-coupled receptor (GPCR) that is the functional core of the receptor as it comprises most of the ligand binding site and couples to the stimulatory heterotrimeric Gs protein. This leads to activation of adenylyl cyclase and production of cAMP. RAMP1 is an accessory subunit that determines CLR ligand selectivity. RAMP1 confers selectivity for CGRP, whereas CLR complexes with either of two other RAMPs, RAMP2/3, are receptors for two adrenomedullin peptides, AM and AM2/IMD.^15^ Both receptor subunits have an N-terminal extracellular domain (ECD) followed by a transmembrane domain (TMD). The CLR TMD spans the membrane with 7-TM helices, whereas RAMP1 has a single TM helix.

Class B GPCRs bind their peptide ligands in a “two-domain” mechanism that is thought to occur in two steps.^16−18^ In the first step, the C-terminal half of the peptide binds to the receptor ECD in a reaction that is typically characterized by moderate affinity (μM *K*_d_). In the second step, the N-terminal half of the peptide binds and activates the receptor TMD, thereby promoting coupling to G protein. In the absence of the ECD interaction, the peptide affinity for the TMD is weak, but full two-site binding is characterized by a strong affinity (nM *K*_d_). As for many GPCRs, class B GPCR peptide agonists typically have higher affinity for the receptor in the presence of G protein,^18,19^ although the extent of this allosteric G protein effect varies depending on the receptor–ligand pair. As the peptide N-terminus activates the receptor, N-terminal truncation typically generates competitive antagonists.

Structural, biochemical, and pharmacological studies support the two-domain model for the CGRPR. CGRP engages the CLR ECD and TMD in the CGRPR active state complex with bound G protein.^20−22^ A cryo-EM structure of the CGRP-bound CGRPR in the absence of G protein showed that CGRP was predominantly single-site engaged to the ECD, and the CLR TMD was in the inactive state.^23^ This suggested that G protein binding plays a critical role in the CGRP N-terminus fully engaging and activating the TMD in the second step. Truncation of the CGRP N-terminus leads to a loss of signaling, as in the traditional CGRP(8-37) antagonist.^24^ Despite these advances, we lack a quantitative understanding of the two-domain mechanism for the CGRPR.

We previously engineered a CGRP variant with four amino acid substitutions (N31D/S34P/K35W/A36S) near the C-terminus that increased its binding affinity ∼1000-fold.^25^ In the truncated (27-37) ECD-binding fragment, this variant was a potent antagonist with single digit nanomolar affinity.^25,26^ The (8-37) version with the potential for two-site ECD/TMD binding exhibited insurmountable antagonism in a functional cAMP accumulation assay, which could be indicative of a slow off-rate/long residence time.^26^ Fitting these data to a hemiequilibrium operational model estimated that it had picomolar affinity and an extremely long residence time of ∼12 h. However, the accuracy of these values was questionable because they came from an indirect functional assay conducted at a single time point. In the (1-37) agonist backbone, the variant had a similar signaling potency to wildtype CGRP at the CGRPR, however, it uniquely maintained high cAMP levels after agonist washout, suggestive of a long residence time.^26^ We termed the agonist variant ssCGRP for sustained signaling (ss). The agonist and truncated antagonist versions of the ss variant may have value as long-acting therapeutics, however, we lack a thorough understanding of their equilibrium and kinetic properties.

The purpose of this study was to quantitatively characterize the CGRPR two-domain mechanism and define the equilibrium and kinetic properties of the ss variant peptides in binding and signaling assays. An additional goal was to apply newer bioluminescence resonance energy transfer (BRET) and biosensor technologies^27,28^ for real-time CGRPR peptide binding and signaling assays in lieu of traditional radioligand binding and end point cAMP accumulation assays. Last, we present a mathematical reaction network model of CGRPR agonist peptide and G protein binding that is consistent with our CGRP binding assay observations and provides novel insights into the relationship between peptide binding affinity and sensitivity to the G protein allosteric effect.

## Materials and Methods

### Cell Culture

COS-7 (African green monkey kidney fibroblast-like cell line, male; CRL 1651) were from American Type Culture Collection (Manassas, VA). Cells were cultured in Dulbecco’s modified Eagle’s medium (DMEM with 4.5 g/L glucose and l-glutamine and 110 mg/L sodium pyruvate) from Gibco (11995-081) with 10% v/v fetal bovine serum (Gibco 16000-044). Cells were grown at 37 °C, 5% CO_2_ in a humidified incubator and passaged twice per week.

### Plasmids

The human CLR and RAMPs were used throughout. Wild-type CLR and RAMP expression plasmids were obtained from cDNA.org. The N-terminally Nanoluciferase (NLuc)-tagged CLR (pcDNA3.1(+)sec/NanoLuc-GSGIS-CLR.23-461) and CAMYEL biosensor plasmids were previously described.^29^

### Synthetic Peptides

Synthetic human peptides αCGRP(1-37), αCGRP(8-37), and AM(13-52) were purchased from Bachem (Bubendorf, Switzerland). AM2/IMD(8-47)-TAMRA, CGRP(27-37), ssCGRP(27-37), ssCGRP(8-37), ssCGRP(1-37), and ssAM(22-52) were previously described.^25,26,29^ Custom peptides synthesized and HPLC-purified by RS Synthesis (Louisville, KY) or Biosynth (Gardner, MA) for this study are as follows: αCGRP(27-37) N31D/S34P/K35-TAMRA/A36S/F37Y (CGRP(27-37)*-TAMRA), αCGRP(8-37) N26K-TAMRA/N31D/S34P/K35W/A36S (ssCGRP(8-37)-TAMRA), and AM(13-36). All peptides were C-terminally amidated. The lyophilized powders were resuspended at 10 mg/mL in sterile ultrapure water. Concentrations of the peptides were determined by UV absorbance at 280 nm with dilutions in 10 mM Tris-HCl, 1 mM EDTA at pH 8.0. Extinction coefficients were calculated from Tyr, Trp, and cystine content. The concentration of αCGRP(1-37) and αCGRP(8-37) were determined by the peptide content reported from Bachem. Peptides were stored at −80 °C with small volume aliquots to limit the number of freeze–thaws.

### NLuc-CLR:RAMP1 Membranes Preparation

Membrane preparation was performed as previously described.^29^ COS-7 cells in ten 150 mm^2^ plastic culture dishes (Corning; 430599) were transiently transfected with 2 μg NLuc-CLR, 2 μg RAMP1, and 46 μg empty pcDNA3.1(+) expression plasmids/dish with 75 μg Polyethylenimine (PEI)/dish. After 2 days, the cells were washed with PBS and harvested with ice-cold PBS + 5 mM EDTA. The cells were pelleted in a centrifuge at 1000*g* for 5 min at 4 °C and resuspended in a hypotonic buffer (25 mM NaHEPES pH 7.5, 2 mM MgCl_2_, 1 mM EDTA, and 1× EDTA-free PIERCE protease inhibitor tablet). After resuspension, cells were homogenized with an Ultra Turrax for 30 s at 10,000 rpm followed by a 10 min incubation. Cell debris was pelleted at 800*g* for 10 min and the supernatant was transferred to ultracentrifuge tubes and centrifuged at 100,000*g* for 1 h. Membranes were resuspended in a wash buffer (25 mM NaHEPES pH 7.5, 250 mM NaCl, 2 mM MgCl_2_, 1 mM EDTA, and 1× protease inhibitor tablet). The homogenization and ultracentrifugation were repeated once more and the membranes were resuspended in storage buffer (25 mM NaHEPES pH 7.5, 25 mM NaCl, 2 mM MgCl_2_, 10% v/v glycerol, and 1× protease inhibitor tablet). The resuspended membranes were homogenized, aliquoted, and flash frozen in liquid nitrogen for storage at −80 °C. Total protein was quantitated using the DC protein assay (BioRad) per the manufacturer’s instructions. The concentration of NLuc-CLR was determined by comparing the membrane prep luminescence to a standard curve using purified NLuc enzyme (Promega; G9711). The total protein concentration was 0.91 mg/mL and the NLuc-CLR was 7.35 nM.

### nanoBRET Ligand Binding Assays

Each binding assay format used NLuc-CLR:RAMP1 membranes at a concentration of 0.01 mg/mL total protein (0.08 nM Nluc-CLR) in a binding buffer of 25 mM NaHEPES pH 7.4, 104 mM NaCl, 5 mM KCl, 1 mM KH_2_PO_4_, 3 mM MgSO_4_, 2 mM CaCl_2_, 1 mg/mL FAF-BSA, and 50 μg/mL saponin. The equilibrium assays were performed at room temperature and the kinetic assays were at 25 °C. For equilibrium saturation binding to characterize the probes, 3-fold serial dilutions of CGRP(27-37)*-TAMRA, ssCGRP(8-37)-TAMRA, or AM2/IMD(8-47)-TAMRA were incubated in a 96-well white plate (Corning; Costar 3912) at a total volume of 50 μL with the membranes and either 50 μM GTPγS for G protein uncoupled state or 30 μM purified sumo-miniGs for the G protein coupled state. H_6_-sumo-miniGs was expressed and purified as previously described.^22^ Reactions were incubated for 2 h (uncoupled state) for CGRP(27-37)*-TAMRA, 2 h (uncoupled state) or 4 h (coupled state) for AM2/IMD(8-47)-TAMRA, and 4 h (uncoupled state) for ssCGRP(8-37)-TAMRA. Furimazine substrate (Promega) was added at 1× per manufacture’s instruction 30 min after the start of the binding reaction. Emission was read with 460 and 610 nm long pass filters in a PolarSTAR Omega plate reader (BMG Labtech). To determine the *K*_d_, agonist concentration was plotted against the 610/460 BRET ratio and fit to a one-site specific binding model in GraphPad Prism (https://www.graphpad.com/guides/prism/latest/curve-fitting/reg_one_site_specific.htm) after subtracting the buffer control (membranes alone). Nonspecific binding was tested for the three highest concentrations of probe using 30 μM unlabeled ssCGRP(1-37) competitor. Nonspecific binding was negligible, so no correction was needed. For display purposes we show the semilog plots for the saturation binding curves (https://www.graphpad.com/guides/prism/latest/curve-fitting/reg_classic_dr.htm).

For equilibrium competition assays, the probe, unlabeled peptide (3-fold serial dilutions), and membranes were mixed and incubated for 2 h for (27-37) peptides and telcagepant or 8 h for (8-37) and (1-37) peptides in a total assay volume of 50 μL. Furimazine was added at 1× according to manufacturer’s directions 30 min after initiating the binding reaction for (27-37) peptides or 3.5 h for the (8-37) and (1-37) peptides. Ten nM CGRP(27-37)*-TAMRA probe was used for (27-37) peptides and telcagepant. Thirty nM AM2/IMD(8-47)-TAMRA probe with 50 μM GTPγS (G protein uncoupled state), or 3 nM AM2/IMD(8-47)-TAMRA probe with 30 μM sumo-miniGs (G protein coupled state) were used for the (8-37) and (1-37) peptides. The BRET 610/460 ratio data (membranes only control subtracted) were plotted against unlabeled ligand concentration and fit to a one-site log IC_50_ competition binding equation in GraphPad Prism (https://www.graphpad.com/guides/prism/latest/curve-fitting/reg_one_site_competition_ic50.htm). The Cheng-Prusoff correction^30^ was used to calculate the K_i_ from the IC_50_.

For association kinetics, 40 μL of 2-fold serial dilutions of CGRP(27-37)*-TAMRA, AM2/IMD(8-47)-TAMRA, or ssCGRP(8-37)-TAMRA in binding buffer were added to a 96 well white plate (Corning; Costar 3912). The membranes were incubated with furimazine for 5 min and then loaded into the plate reader injector. 40 μL of the membranes were injected into the peptide serial dilutions in the plate for a total volume of 80 μL. Emission at 460 and 610 nm were read every 16 s for 40 min for CGRP(27-37)*-TAMRA and AM2/IMD(8-47)-TAMRA and 1 h and 15 min for ssCGRP(8-37)-TAMRA with the PolarSTAR Omega plate reader mixing 300 rpm for 1 s before each cycle. The BRET ratio 610/460 was plotted against time with background subtraction (membranes alone) and each curve was fit to a one-phase association exponential model in GraphPad Prism (https://www.graphpad.com/guides/prism/latest/curve-fitting/reg_exponential_association.htm) using the first 10 min of data to minimize the effects of signal decay or the full hour and 15 min for ssCGRP(8-37)-TAMRA, which exhibited less signal decay. The signal decay varied depending on the probe used in the assay. For the CGRP(27-37)*-TAMRA probe and AM2/IMD-TAMRA probe, the signal decay became more pronounced after 10 min. For ssCGRP(8-37)-TAMRA probe, little signal decay was seen in association kinetic experiments, but signal decay was observed in the dissociation kinetic experiments that had a much longer assay duration. The observed association rates (*k*_obs_) were plotted against probe concentration and the data was analyzed by linear regression. The *k*_on_ and *k*_off_ were obtained as the slope and y-intercept, respectively. The *K*_d_ (*k*_off_/*k*_on_), half-life (ln 2/*k*_off_), and residence time (1/*k*_off_) values were calculated.

For competition association kinetics, assays were performed similar to the association kinetics with 3-fold serial dilutions of the unlabeled peptide made in binding buffer with 20 nM CGRP(27-37)*-TAMRA for unlabeled (27-37) peptides. Emission at 460 and 610 nm were measured every 16 s for 45 min with the PolarSTAR Omega plate reader mixing 300 rpm for 1 s before each cycle. The curves were fit using GraphPad Prism to the Motulsky-Mahan competition kinetics equation^31^ modified to account for the signal decay component as previously described^32^ to determine the *k*_on_ and *k*_off_ of the competitor. The *K*_d_ (*k*_off_/*k*_on_), half-life (ln 2/*k*_off_), and residence time (1/*k*_off_) values were calculated.

For dissociation kinetics, the membranes and probe in binding buffer were incubated in a 96-well white plate (Corning; Costar 3912) followed by furimazine addition at 1× for 5 min (75 μL volume). Emission at 460 and 610 nm were read every 13 s for 5 min with mixing 300 rpm for 1 s before each cycle to establish a baseline prior to injection of unlabeled competitor to initiate the dissociation phase or buffer control (25 μL volume). The buffer control defined the stability of the signal. For ssCGRP(8-37)-TAMRA, the membranes and 1 nM probe final were preincubated for 30 min before furimazine addition. Unlabeled ssCGRP(8-37) (4×; 10 μM) or binding buffer was loaded into the injectors and injected into the plate, which was read for 4 h. For AM2/IMD(8-47)-TAMRA, the membranes and 10 nM probe final were preincubated for 15 min before furimazine addition. ssCGRP(8-37) (4×; 1 μM) or binding buffer was loaded into the injectors and injected into the plate, which was read for 1 h. The BRET ratio 610/460 was plotted against time and the membrane only control was subtracted. The dissociation data were normalized to their corresponding buffer control injections as 100% (100*value/baseline) to account for signal decay with time. The normalized dissociation curves were fit to a one-phase (dashed line) (https://www.graphpad.com/guides/prism/latest/curve-fitting/reg_exponential_decay_1phase.htm) or a two-phase (solid line) (https://www.graphpad.com/guides/prism/latest/curve-fitting/reg_exponential_decay_2phase.htm) exponential decay models in GraphPad Prism to determine the *k*_off_. The half-life (ln 2/*k*_off_) and residence time (1/*k*_off_) values were calculated.

### Native PAGE Thermostability Assay

This assay and the MBP-CLR-EGFP:MBP-RAMP1 (MBP: Maltose Binding Protein; EGFP: Enhanced Green Fluorescent Protein) membrane preparation were previously described.^22,29^ In brief, the membranes were incubated with peptides for 30 min on ice followed by Lauryl Maltose Neopentyl Glycol (LMNG)/ Cholesteryl Hemisuccinate (CHS) solubilization for 2 h at 4 °C. After centrifugation, the supernatants were incubated at various temperatures for 30 min. The samples were then centrifuged, and the supernatants analyzed by native PAGE with imaging for EGFP fluorescence using the BioRad Chemidoc MP imager. Densitometry was performed using Image Lab software v 6.1 as described^33^ and the band volumes were plotted against temperature in GraphPad Prism and fit to a sigmoidal dose–response with variable slope equation (https://www.graphpad.com/guides/prism/latest/curve-fitting/reg_classic_dr_variable.htm). The EC_50_ provides an empirical melting temperature.

### LANCE cAMP Accumulation Assay

cAMP accumulation assays were performed as previously described.^26^ In brief, COS-7 cells were seeded in a 96-well clear plate (Corning; Costar 3997) with a density of 20,000 cells/well, 100 μL/well, and incubated at 37 °C and 5% CO_2_ for 24 h. Cells were transiently transfected with 125 ng receptor and 125 ng RAMP with 375 ng branched PEI and incubated for 48 h at 37 °C and 5% CO_2_. Cells were stimulated with serial dilutions of the agonist for 15 min at 37 °C in the presence of 1 mM 3-Isobutyl-1-methylxanthine (IBMX). Cells were lysed and cAMP accumulation was measured using the LANCE kit from PerkinElmer per manufacturer’s instructions.

### CAMYEL cAMP Biosensor Competition Assay with N-Terminal TMD-Binding Agonist

COS-7 cells were seeded in a 96-well white plate (Corning; Costar 3917) with a density of 20,000 cell/well, 100 μL/well, and incubated at 37 °C and 5% CO_2_ for 24 h. Cells were transiently transfected with 250 ng DNA total with 25 ng CLR, 25 ng RAMP, 125 ng CAMYEL biosensor, 75 ng empty pcDNA3.1(+) vector, and 375 ng branched PEI per well. Transfected cells were incubated at 37 °C and 5% CO_2_. Two days after transfection, cells were washed with 1× PBS and preincubated for 30 min at room temperature in assay buffer 25 mM NaHEPES pH 7.4, 104 mM NaCl, 5 mM KCl, 1 mM KH_2_PO_4_, 1.2 mM MgSO_4_, 2 mM CaCl_2_, 1 mg/mL fatty-acid-free bovine serum albumin (FAF-BSA), and 5 mM glucose. The preincubation was followed by addition of coelenterazine h (10 μM) and simultaneous addition of 30 μM AM(13-36) agonist and antagonist as indicated, and incubated for 1 h at room temperature in total volume of 50 μL. Emissions at 475 and 535 nm were read in a PHERAstar FS plate reader (BMG Labtech, Ortenberg, Germany). The buffer control (no ligand) was subtracted from each data set and plotted with the corresponding 475/535 BRET ratio in GraphPad Prism.

### CAMYEL cAMP Biosensor Assay with Agonist Stimulation Followed by Antagonist Challenge

This assay was performed as previously described using COS-7 cells.^29,34^ Cells were seeded at 20,000 cells/well and 100 μL/well in a 96-well white plate (Corning; Costar 3917) and incubated at 37 °C and 5% CO_2_ for 24 h. The cells were transiently transfected with 25 ng CLR, 25 ng RAMP, 125 ng CAMYEL biosensor plasmid, and 75 ng empty pcDNA3.1 using 375 ng branched PEI per well. Cells were incubated 2 days at 37 °C and 5% CO_2_. Assays were performed at room temperature in buffer containing 25 mM NaHEPES pH 7.4, 104 mM NaCl, 5 mM KCl, 1 mM KH_2_PO_4_, 1.2 mM MgSO_4_, 2 mM CaCl_2_, 1 mg/mL FAF-BSA, and 5 mM glucose. Cells were washed with PBS, preincubated with buffer for 30 min, and then stimulated with 100 nM agonist in the presence of 10 μM coelenterazine h (NanoLight) and in a total volume of 50 μL. After 15 min, 5 μL of buffer control or 10 μM antagonist challenge was added using ssCGRP(8-37) for CLR-RAMP1 or ssAM(22-52) for CLR-RAMP2/3. Emission at 475 and 535 nm were measured every 10 s for 100 min in the PolarSTAR Omega plate reader. The BRET ratio of 475/535 was plotted against time. The no peptide baseline control was subtracted from each data set. The decay phase after antagonist addition was fit to a one-phase exponential decay model in GraphPad Prism with the Y_0_ value constrained to be equal to the last point on a linear regression line fit to the data 5 min before antagonist addition.

### Statistics

For each assay, duplicate technical replicates were used and data points are shown as mean ± SD. The figures show representative plots from a single independent experiment unless otherwise stated in the figure legend. All experiments were performed with at least three independent replicates (*n* = 3) on different days and the parameters of interest (e.g., *K*_d_, *K_i_*, *k*_on_, *k*_off_, *t*_1/2_) were reported as mean ± SEM. Statistical analyses were performed in GraphPad Prism with one-way ANOVA and Tukey’s post hoc test with a confidence interval of 95% reaching statistical significance of *p* < 0.05 comparing the mean of the three independent replicates.

### Reaction Network

The reaction network describing the activation mechanism of the CLR:RAMP1 complex upon CGRP peptide and G protein binding is illustrated in Figure 8A. To model the reaction network, we considered a system involving 12 species. These species represent different conformational states of CLR:RAMP1 complex and its interactions with CGRP peptide and G proteins. Each reversible reaction between species is shown by an arrow and labeled with its equilibrium constant (*K*) that governs the ratio between the product and reactant concentrations:
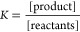
1For the activation reactions, the product state is the activated state (e.g., *K*_act1_ = CLR_act_/CLR_inac*t*_). For the binding reactions, the product state is the bound state (e.g., *K*_g-bind1_ = G:CLR_act_/(CLR_inact_*G*)*, *K*_ecd-bind_ = CLR_act_:CGRP_ecd_/(CLR_act_*CGRP) and *K*_tmd-bind1_ = CLR_act_:CGRP_full_/CLR_act_:CGRP_ecd_).

In our model, we made the assumption that RAMP1 constantly associated with CLR, and therefore, CLR:RAMP1 receptor complex is referred to as CLR. The inner loop of the network reflects the reactions between different conformational states of CLR and G protein interactions. The model accounts for CLR existing in both active (CLR_act_) and inactive (CLR_inact_) conformations, with G proteins capable of binding to either conformation to form the G:CLR_act_ and G:CLR_inact_ complexes. The transitions between these four states are governed by four equilibrium constants in which *K*_act1_ describes the equilibrium between CLR_act_ and CLR_inact_ (*K*_act1_ = CLR_act_/CLR_inact_), *K*_act3_ describes the equilibrium between G:CLR_act_ and G: CLR_inact_ (*K*_act3_ = G:CLR_act_/G:CLR_inact_), *K*_gbind1_ governs the binding of G protein to CLR_act_ (*K*_g-bind1_ = G:CLR_act_/CLR_act_), and *K*_g-bind2_ governs the binding of G protein to CLR_inact_ (*K*_g-bind1_ = G:CLR_act_/CLR_inact_). We note that conservation of energy for any closed loop in the reaction network is enforced by “loop closure” rules, where the product of the equilibrium constants going around a loop must be equal to 1. In the case of the inner loop, this is enforced by ensuring *K*_act1_/*K*_act3_ = *K*_g-bind2_/*K*_g-bind1_.

This inner loop of the network is extended by introducing the ECD-binding segment of CGRP ligand (CGRP_ecd_) binding to the CLR. CGRP binds to its receptor with a two-step, two-domain mechanism. First, the ECD-binding segment of CGRP (denoted as CGRP_ecd_) binds to the ECD of CLR, followed by the TMD-binding segment of CGRP, which binds to the CLR TMD. This is strictly enforced in the model, meaning the population of CLR that is bound only through the TMD-binding segment of CGRP is considered to be zero. The binding affinity of CGRP_ecd_ also does not depend on the activation state of CLR or the presence of G protein since the ECD-binding segment is structurally isolated from the TMD-binding segment. This results in having the same equilibrium constant, denoted as *K*_ecd-bind_, governing the binding of CGRP_ecd_ to CLR_act_ (CLR_act_:CGRP_ecd_), CLR_inact_ (CLR_inact_:CGRP_ecd_), G:CLR_act_ (G:CLR_act_:CGRP_ecd_), and G:CLR_inact_ (G:CLR_inact_:CGRP_ecd_). This has some additional consequences in the reaction network due to loop closure rules mentioned above. For example, the same activation constant between CLR_inact_ and CLR_act_ (*K*_act1_) must also be used for CLR_inact_:CGRP_ecd_ and CLR_act_:CGRP_ecd_.

The outer loop represents the final stage of the reaction network where the TMD-binding segment of CGRP also binds to CLR resulting in full ligand binding, denoted as CGRP_full_. In our model, while going from CGRP_ecd_ to CGRP_full_, the binding affinity of the CGRP TMD-binding segment only depends on the *CLR* activation state, and not on G protein bound status since the CGRP TMD-binding segment does not directly interact with the G protein binding site. Because of this assumption, the binding of the TMD-binding segment of CGRP to CLR_act_:CGRP_ecd_ and G:CLR_act_:CGRP_ec*d*_ are both governed by the *K*_tmd-bind1_ equilibrium constant, whereas the binding of the CGRP TMD-binding segment to CLR_inact_:CGRP_ecd_ and G:CLR_inact_:CGRP_ecd_ are both governed by *K*_tmd-bind2_ equilibrium constant. In addition, due to loop closures, the following restraints are needed in the reaction network:
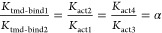
2
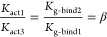
3where α is the activation enhancement factor and β is the G protein activation enhancement factor.

### Solving the Reaction Network

All equilibrium constants defined in the reaction network were used to solve the system of equations that governs the equilibrium distribution of species. Our system is overdetermined, and it has more equations than unknowns, thereby, a minimal set of equations involving each network quantity described above as well as the total concentration restraints for G protein, CLR, and CGRP are used. The overview of the quantities used in equations is given in Table 1.

**Table 1:** Overview of the Species and Their Notations Used in Equations

**species**	**description**	**notation**
CLR_inact_	the inactive form of CLR	*x*_0_
CLR_act_	the active form of CLR	*x*_1_
CLR_inact_:CGRP_ecd_	the inactive form of CLR with the ECD-binding segment of CGRP bound	*x*_2_
CLR_act_:CGRP_ecd_	the active form of CLR with the ECD-binding segment of CGRP bound	*x*_3_
CLR_inact_:CGRP_full_	the inactive form of CLR with the CGRP fully bound	*x*_4_
CLR_act_:CGRP_full_	the active form of CLR with the CGRP fully bound	*x*_5_
G:CLR_inact_	the inactive form of CLR with G protein bound	*x*_6_
G:CLR_act_	the active form of CLR with G protein bound	*x*_7_
G:CLR_inact_:CGRP_ecd_	the inactive form of CLR with the ECD-binding segment of CGRP and G protein bound	*x*_8_
G:CLR_act_:CGRP_ecd_	the active form of CLR with the ECD-binding segment of CGRP and G protein bound	*x*_9_
G:CLR_inact_:CGRP_full_	the inactive form of CLR with the CGRP fully and G protein bound	*x*_10_
G:CLR_act_:CGRP_full_	the active form of CLR with the CGRP fully and G protein bound	*x*_11_
G	free G protein	*x*_12_
CGRP	free CGRP peptide	*x*_13_
G_tot_	total concentration restraint for G protein	G_tot_
CGRP_tot_	total concentration restraint for CGRP peptide	CGRP_tot_
CLR_tot_	total concentration restraint for *CLR*	CLR_tot_

The minimal set of reaction equations, together with the total concentration restraints, gives the following 14 equations with 14 unknowns:
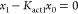

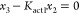

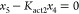

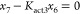

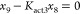

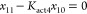

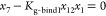

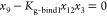

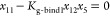

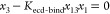

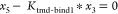

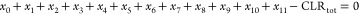

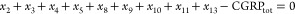

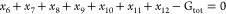


It is not possible to use linear algebra to solve these equations due to the four nonlinear equations, but we can simplify these equations considerably by eliminating *x*_0_, *x*_2_, *x*_4_, *x*_6_, *x*_8_, *x*_10_ and *x*_5_. This will leave us with 7 equations with 7 unknowns:
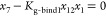

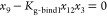

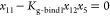

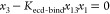

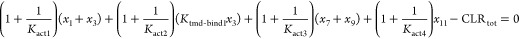

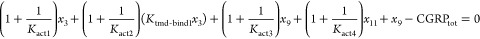

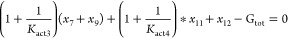


If we simplify these equations further by eliminating *x*_7_, *x*_9_, *x*_11_:
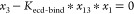

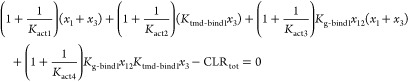

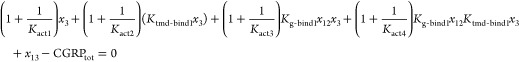

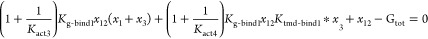


As a last step, we simplify the equations by eliminating *x*_3_ and obtain 3 equations with 3 unknowns:
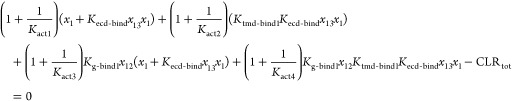

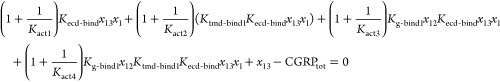

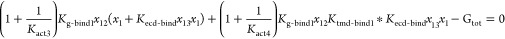
These can be efficiently solved using a nonlinear equation solver such as fsolve from the scipy python package.^35^

### Reaction Network Parameters

There are 9 total parameters to be set for this model: (I) Equilibrium constant of CGRP_ecd_ binding (*K*_ecd-bind_), (II) equilibrium constant of the TMD-binding segment of CGRP binding to activated CLR (*K*_tmd-bind1_), (III) baseline activation ratio (*K*_act1_), (IV) G protein binding affinity to activated CLR (*K*_g-bind1_), (V) total concentration of G protein (*G*_tot_), (VI) total concentration of CGRP (CGRP_tot_), (VII) total concentration of CLR (CLR_tot_), (VIII) the activation enhancement factor (α), (IX) the G protein activation enhancement factor (β). The remaining parameters can then be determined as follows:
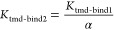
4
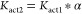
5
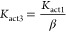
6
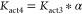
7
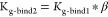
8Where possible, we used relevant experimental data to fix the values of parameters in the model, as described below. For parameters where this was not possible, we made educated guesses for reasonable values and then evaluated the qualitative predictions of the model over a wide range of possible values. Based on *in vitro* experiments presented in this paper, the ECD-binding segment of CGRP and ssCGRP binding affinities to CLR are set to 10^–6^ and 10^–9^ mol/L, respectively, indicating a stronger binding affinity for ssCGRP. The equilibrium constant of the TMD-binding segment of CGRP binding to activated CLR representing the ratio of bound to unbound TMD-binding segment of CGRP in the active state of CLR is set to 7500. This was chosen to match the binding affinities of full-length CGRP both with and without G protein as closely as possible. This is consistent with cryo-EM evidence that in the absence of G protein, only a small percentage of CLR has TMD-binding segment of CGRP bound.^23^ The TMD-binding equilibrium constant is further scaled by the activation enhancement factor (α = 1000) to account for reduced binding of CGRP to the TMD binding when CLR is in inactive state (eq 4). Activation of CLR is governed by *K*_act1_ which is set to 0.00025 (which by default favors the inactive state) and this is also scaled by the same α value to reflect the enhanced activation state of CLR in the presence of CGRP (eq 5). When G protein is bound, the activation is even stronger and this further increase in activation is reflected in *K*_act3_ = *K*_act1_/β, where β is set to 10^–5^ (eq 6). This can also be further scaled by α to describe the highest level of activation when both CGRP/ssCGRP and G protein are bound to CLR (eq 7). The G protein binding equilibrium constant, *K*_g–bind1_, was based on a native PAGE mobility shift assay that showed ∼200 nM binding affinity for G protein in the presence of saturating agonist.^22^*K*_g–bind1_ is thus set to 0.005 nM^–1^ and this is scaled by β to reflect weaker binding to inactive CLR (eq 8). Under our experimental conditions, the total concentration of G protein was 30 μM and total CLR was 0.08 nM. These were used by default in our models, unless otherwise stated. For the total concentration of CGRP/ssCGRP, a range of values between 10^–10^–10^–5^ mol/L are used.

## Results

**Figure 1:**
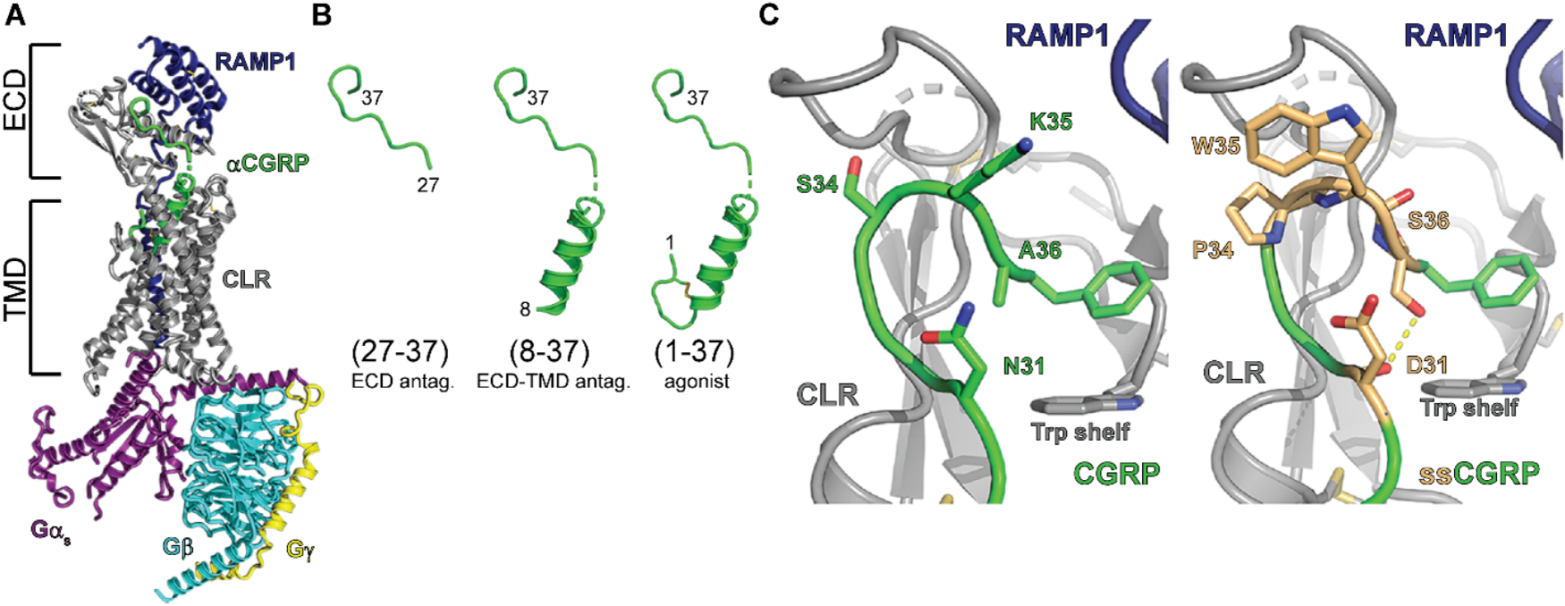
Structural Depiction of the different lengths of peptides and mutations in ssCGRP. (A) Structural depiction of CGRPR bound to αCGRP and G protein. (B) Structural depiction of the different lengths of peptides used throughout these studies. (C) The C-terminus of CGRP bound to CGRPR ECD (left). Model of the ssCGRP mutations (tan) in the C-terminus of the peptide bound to CGRPR ECD (right). All structural depictions were modeled and modified from PDB 6E3Y.

To characterize the CGRPR two-domain mechanism (Figure 1A), we examined the binding and/or signaling properties of peptides of three different lengths (Figure 1B) for both the wildtype CGRP and the ssCGRP variant (Figure 1C). These included the (27-37) antagonist fragment that binds only the receptor ECD, and the (8-37) antagonist fragment and full-length (1-37) agonist, which both have the potential for two-site ECD/TMD binding. The assays employed included nanoBRET receptor binding assays^28^ in equilibrium and kinetic formats, and functional cAMP signaling assays using the CAMYEL cAMP biosensor,^27^ which enables both end point and real-time kinetic measurements of cAMP. The receptor binding assays used a membrane preparation from COS-7 cells cotransfected with N-terminally nanoluciferase (Nluc)-tagged CLR and RAMP1. Nluc-CLR:RAMP1 displayed normal cAMP signaling pharmacology for CGRP and AM agonists in COS-7 cells (Figure S1), indicating that the Nluc BRET donor on CLR did not alter function of the CGRP receptor. The functional assays used live COS-7 cells cotransfected with wild-type CLR, RAMP1, and the CAMYEL biosensor.

### Characterization of the (27-37) Antagonist Fragments

For nanoBRET peptide binding assays, we designed a TAMRA acceptor fluorophore-labeled variant of CGRP(27-37) as a single-site ECD binding probe to facilitate analyses of peptide binding to the receptor ECD complex. This probe had the N31D/S34P/A36S affinity-enhancing substitutions identified in prior work^25,36^ as well as F37Y^25^ to enable its potential future use for CLR complexes with RAMP2/3. The TAMRA label was on the K35 side chain. Hereafter we refer to this probe as CGRP(27-37)*-TAMRA. Equilibrium saturation binding experiments with the Nluc-CLR:RAMP1 membranes revealed a probe affinity (*K*_d_) of 28.7 nM (Figure S2A; Table S1). Nonspecific binding was negligible as demonstrated by blockage of the BRET signal to baseline by an excess of unlabeled competitor peptide (Figure S2A). Association kinetic experiments were performed with several probe concentrations. Signal decay was observed with extended incubation (Figure S2B), so we limited the curve fitting to the first 10 min, by which point equilibrium was reached (Figure S2C). Each curve was fit to a one-phase association exponential equation and the observed rates were plotted against probe concentration as described^37^ (Figure S2D). This yielded an on rate (*k*_on_) of 1.65 × 10^8^ M^–1^ min^–1^, off rate (*k*_off_) of 3.81 min^–1^, and half-life of 11 s. The calculated *K*_d_ from the kinetic experiments, 22.5 nM, was in good agreement with the equilibrium *K*_d_. The probe *K*_d_, *k*_on_, and *k*_off_ values were used in nanoBRET competition experiments to determine equilibrium and kinetic parameters for unlabeled competitor CGRP(27-37) peptides.

**Figure 2:**
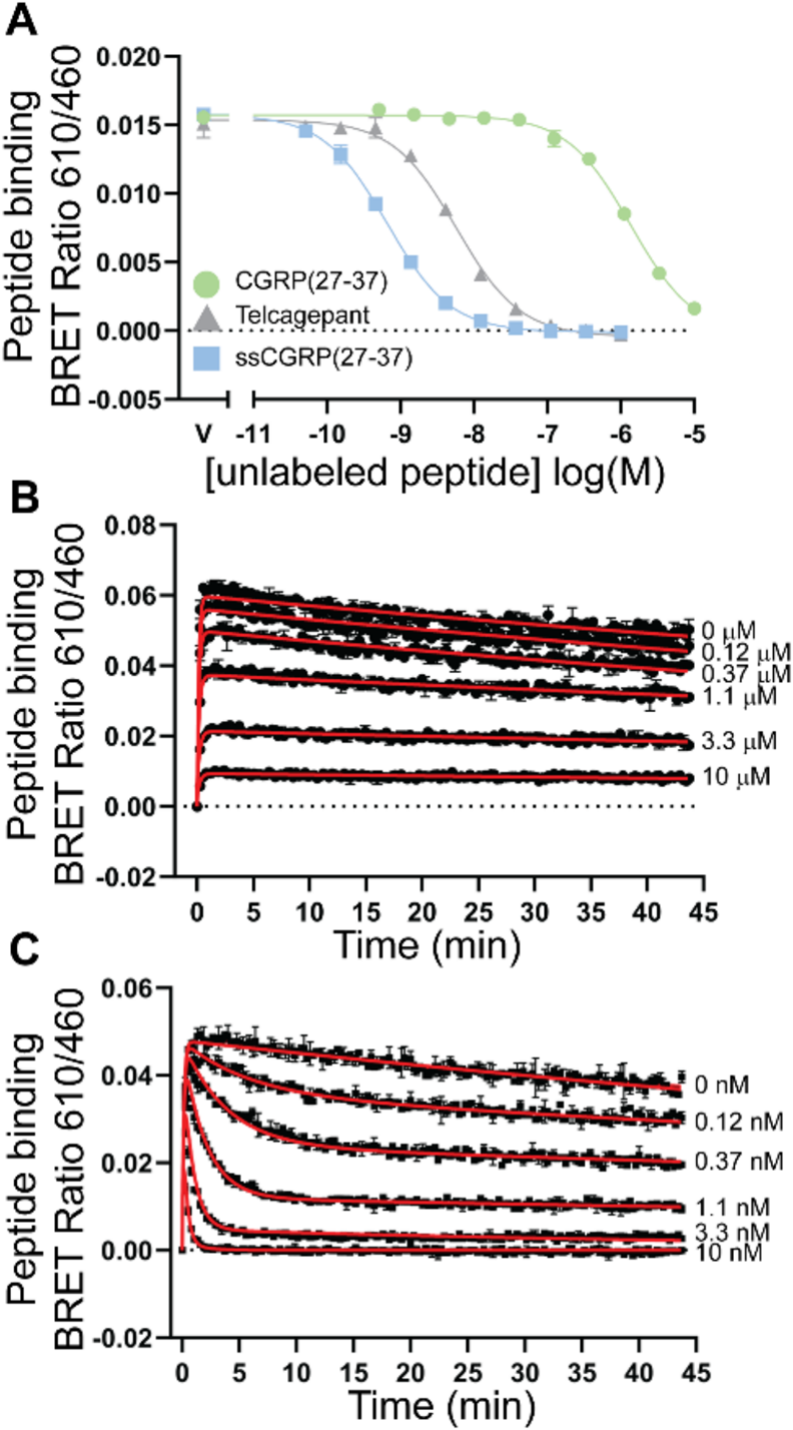
Equilibrium and kinetic binding of CGRP and ssCGRP (27-37) peptides. (A**)** nanoBRET competition equilibrium in COS-7 membranes competing against 10 nM CGRP(27-37)*-TAMRA probe. (B and C) nanoBRET competition simultaneous addition kinetics in COS-7 membranes of CGRP(27-37) (B) or ssCGRP(27-37) (C) competing against 20 nM CGRP(27-37)*-TAMRA probe. Data were analyzed using a Motulsky-Mahan equation that incorporates kinetic drift. The kinetic curve fits are shown in red.

In equilibrium competition binding experiments, CGRP(27-37) had an affinity *K_i_* = 1 μM and the ssCGRP(27-37) affinity was ∼3 orders of magnitude stronger (*K_i_* = 0.53 nM) (Figure 2A and Table S2). The CGRP(27-37) affinity was similar to the affinities of 2-3 μM reported by other groups using a radioligand binding assay with SK-N-MC membranes that have endogenous CGRP receptor.^36,38^ The ssCGRP(27-37) affinity was in good agreement with its ∼1 nM affinity for purified CLR-RAMP1 ECD complex,^26^ and with its 2 nM affinity (apparent p*K*_B_) measured for full-length CLR:RAMP1 in a functional cAMP antagonism assay.^25^ The small molecule telcagepant, which is an antagonist of the CGRPR originally developed as a migraine drug, had a *K_i_* of 4.6 nM (Figure 2A), which is similar to previously reported values.^39,40^ These results indicated that the nanoBRET peptide binding assay yielded CGRPR ligand binding parameters in good agreement with traditional methods and confirmed that the ssCGRP(27-37) variant had ∼1000-fold increased receptor binding affinity.

Motulsky-Mahan (M-M) competition binding kinetics experiments^31^ with simultaneous addition of probe and competitor were used in the nanoBRET assay to define the kinetic parameters of the unlabeled competitor peptides (Figure 2B,C). The M-M equations were supplemented with a decay equation as previously described^32^ to account for the BRET signal decay observed with the CGRP(27-37)*-TAMRA probe. CGRP(27-37) had an on rate of 1.17 × 10^7^ M^–1^ min^–1^ and an off rate of 12.2 min^–1^ that yielded a calculated *K*_d_ of 1 μM and a half-life of 3.6 s (Figure 2B and Table S2). The ssCGRP(27-37) curves displayed the classic rise and fall shape indicative of a slow off-rate competitor. Fitting these yielded an on rate of 1.93 × 10^8^ M^–1^ min^–1^ and an off rate of 0.13 min^–1^ (Figure 2C and Table S2). These rates gave a half-life of 5.2 min and a calculated *K*_d_ of 0.73 nM (Table S2). The calculated *K*_d_ values for both peptides were in good agreement with the *K_i_* values from the equilibrium experiments. Overall, ssCGRP(27-37) had a 16-fold faster on rate and 90-fold slower off rate than wt CGRP(27-37).

### Characterization of the (8-37) Antagonist Fragments

We next performed nanoBRET binding experiments for the (8-37) fragments, which have the potential for two-site ECD/TMD binding. We chose to use a two-site ECD/TMD-binding probe and we settled on the AM2/IMD(8-47)-TAMRA agonist that we previously used for CLR:RAMP3.^29^ Equilibrium saturation binding assays were performed in the presence of GTPγS to uncouple G protein from the receptor and separately in the presence of the purified Gs protein surrogate miniGs^22,41^ to stabilize the receptor in the active G protein-coupled state. Nonspecific binding was again negligible and the probe *K*_d_ was 41 nM for the uncoupled state and 6.5 nM for the coupled state (Figure S3A,B and Table S1). Association binding kinetics experiments in the uncoupled state showed signal decay with extended incubation (Figure S3C), so we limited our curve-fitting analysis to the first 10 min (Figure S3D). This gave an on rate of 6.64 × 10^7^ M^–1^ min^–1^ and an off rate of 0.49 min^–1^ (Figure S3D,E and Table S1). These yielded a calculated *K*_d_ of 8.1 nM and a half-life of 1.6 min. Direct dissociation experiments for the probe in the uncoupled state showed a slight signal drift over time (Figure S3F), so this was corrected for by normalization to the buffer control (Figure S3G). The normalized data yielded a dissociation curve best fit by a two-phase exponential decay. The *k*_off_ fast and slow were 1.3 and 0.11 min^–1^, respectively (Figure S3G and Table S1). These results were similar to our previous observations for this probe at CLR:RAMP3 in that only the direct dissociation experiments revealed two-phase behavior consistent with two-site binding.^29^

**Figure 3:**
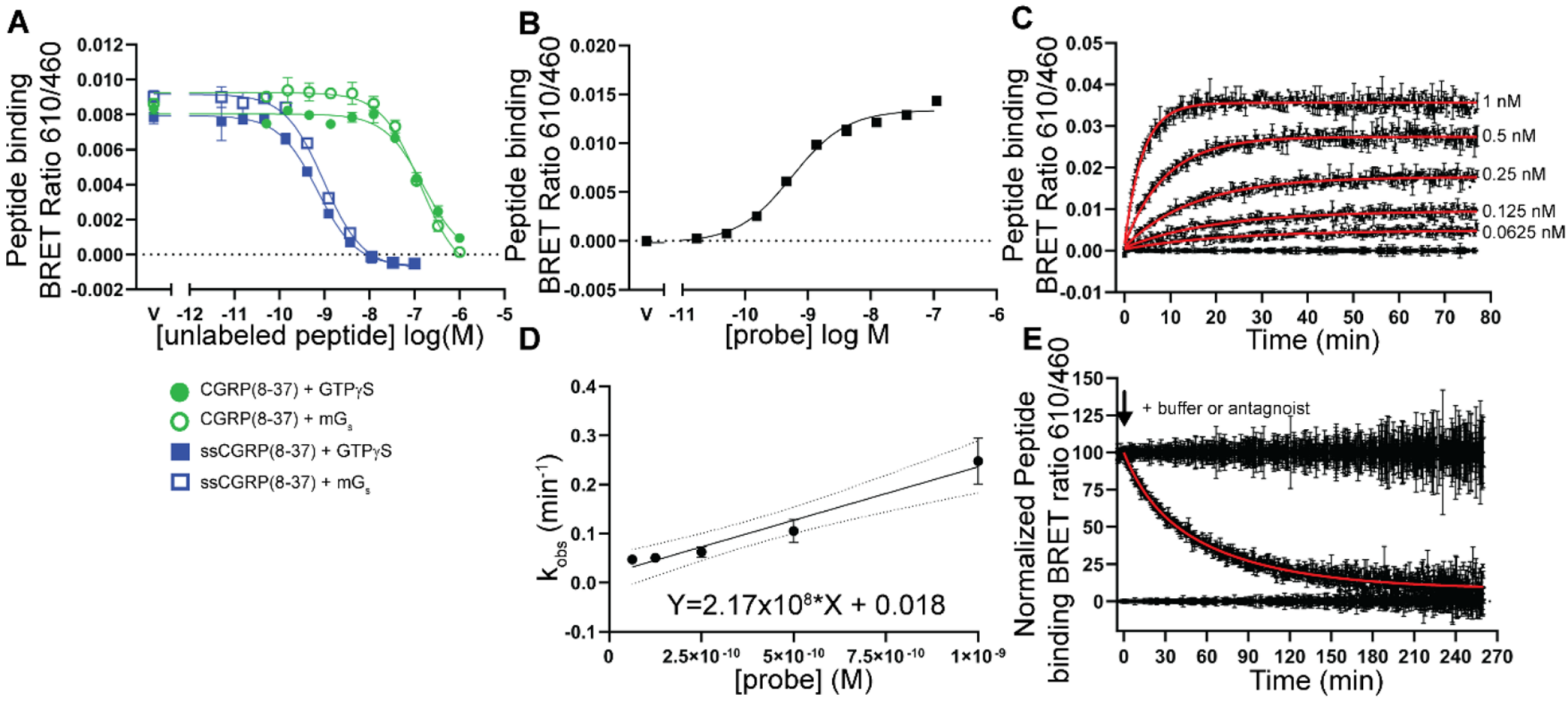
Equilibrium and kinetic binding of CGRP and ssCGRP (8-37) peptides. (A) nanoBRET competition equilibrium in COS-7 membranes competing against 30 nM AM2/IMD(8-47)-TAMRA probe in the G protein uncoupled state (50 μM GTPγS) or 3 nM probe in the G protein coupled state (30 μM mG_s_). (B) Equilibrium binding of ssCGRP(8-37)-TAMRA probe in COS-7 membranes. (C) Association kinetics of ssCGRP(8-37)-TAMRA probe in COS-7 membranes. (D) Linear plot of association rates from C plotted against probe concentration. Plot combines all *n* = 3 independent replicates with mean ± SEM. (E) Dissociation kinetics of 1 nM ssCGRP(8-37)-TAMRA probe in COS-7 membranes. Dissociation was initiated by addition of 10 μM unlabeled ssCGRP(8-37). Kinetic curve fits are shown in red.

Equilibrium competition binding experiments were performed to characterize the affinities of unlabeled CGRP(8-37) and ssCGRP(8-37) for both the uncoupled and coupled receptor states (Figure 3A). CGRP(8-37) bound the uncoupled and coupled states with similar *K_i_* values of 96.7 ± 2.4 and 92.1 ± 1.2 nM, respectively. ssCGRP(8-37) bound the uncoupled and coupled states with similar *K_i_* values of 0.43 ± 0.02 and 0.60 ± 0.05 nM, respectively. These results indicated that neither of these antagonist peptides discriminated the two receptor states and that the ss version of the (8-37) fragment had picomolar affinity that was roughly 200-fold higher than its wildtype counterpart.

To define the kinetic properties of ssCGRP(8-37), we examined the direct binding of a labeled version of this peptide. This was done rather than using the kinetic competition method because the M-M equations were developed assuming a single site binding mechanism. We designed a TAMRA-labeled version of ssCGRP(8-37) with the label on the side chain of a Lys residue substituted for N26. Mutation of N26 had no effect on CGRPR ECD binding^42^ and structural studies indicated that N26 is solvent exposed.^20,21^ In an equilibrium saturation binding assay, nonspecific binding was again negligible (Figure S4A) and ssCGRP(8-37)-TAMRA had a *K*_d_ of 0.4 nM (Figure 3B; Table S3). This was in good agreement with the *K_i_* for unlabeled ssCGRP(8-37) obtained in Figure 3A, indicating that the TAMRA label did not alter the binding affinity of ssCGRP(8-37). Association binding kinetics experiments yielded a *k*_on_ of 2.17 × 10^8^ M^–1^ min^–1^, *k*_off_ of 0.018 min^–1^, calculated *K*_d_ of 0.085 nM, and half-life of 38 min, respectively (Figure 3C,D; Table S3). The association data were best fit to a one-phase association curve, which may not accurately account for potential two-site behavior, so this may explain the discrepancy between the calculated *K*_d_ and the equilibrium saturation binding *K*_d_. For dissociation kinetics, the receptor-probe complex was preformed for 40 min before the dissociation phase was initiated with excess unlabeled ssCGRP(8-37). Signal decay was observed (Figure S4B), so this was corrected for by normalization to the buffer control. The normalized dissociation data were best fit to a two-phase decay curve (Figure 3E). The *k*_off_ fast was 0.053 min^–1^ and *k*_off_ slow was 0.013 min^–1^ with percent fast 25.6% (Figure 3E and Table S3). This yielded half-lives of 13 and 52 min for the fast and slow components, respectively. These data indicated that ssCGRP(8-37) is a slow off-rate antagonist with a long residence time (1/*k*_off_ slow = 76 min; Table S3).

### (8-37) Fragments, but Not (27-37), Antagonize an N-terminal TMD-Binding Agonist

**Figure 4:**
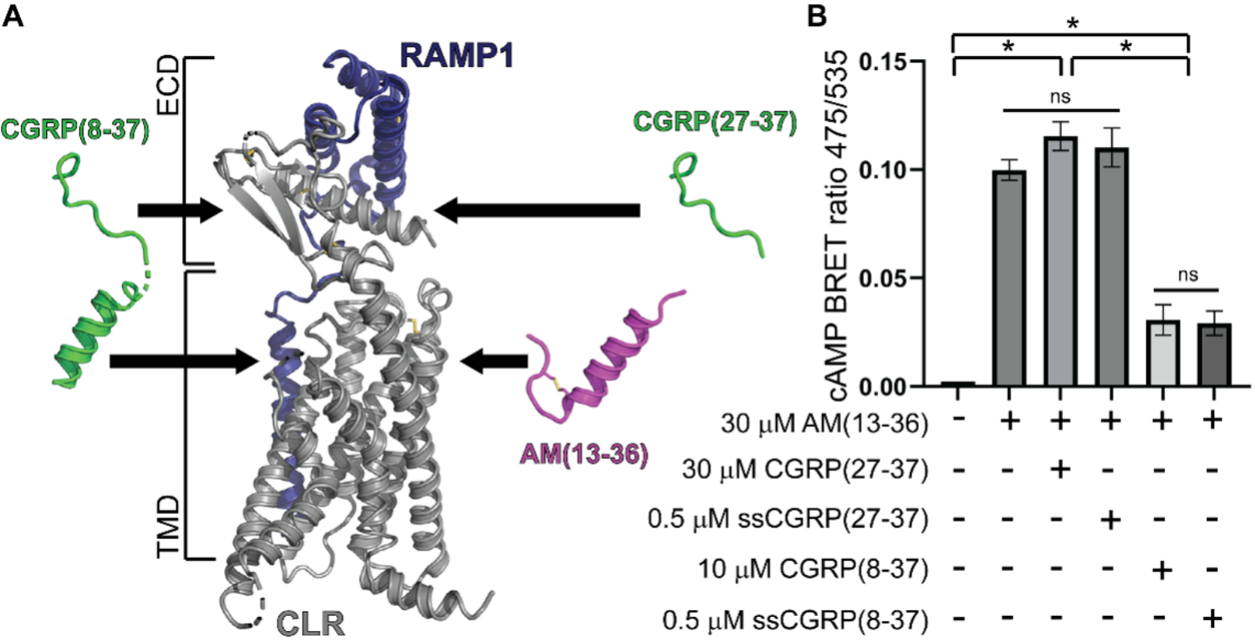
Two-domain binding of CGRP and ssCGRP peptides. (A) Structural depictions of N-terminal agonist cAMP CAMYEL competition assay. (B) N-terminal agonist cAMP CAMYEL competition assay in COS-7 cells. Graph showing combined *n* = 3 independent replicates with mean ± SEM.

For wildtype CGRP, the (8-37) fragment exhibited a 10-fold increased affinity over the (27-37) fragment consistent with it making additional contacts to the TMD, however, these two fragments had similar affinities for the ss variant. This raised the question of to what extent the longer antagonists engage the receptor TMD, particularly considering the cryo-EM structure of the CGRPR in the absence of G protein that showed that the full-length CGRP agonist was predominantly single-site ECD engaged.^23^ To provide insight into this, we compared the ability of the (27-37) and (8-37) fragments to antagonize CGRPR cAMP signaling resulting from a C-terminally truncated AM(13-36) agonist that comprises the TMD-binding portion of the agonist (Figure 4A). In an end point cAMP biosensor assay, we detected AM(13-36) agonism of the CGRPR at a very high concentration (30 μM) (Figure 4B). Both wt and ssCGRP(27-37) failed to antagonize this activity, whereas the two (8-37) versions significantly diminished the activity of AM(13-36), albeit not to baseline (Figure 4B). These results suggested that both wt and ss (8-37) antagonist fragments may engage the receptor TMD.

### CGRP and ssCGRP Effects on Heterodimer Thermostability

**Figure 5:**
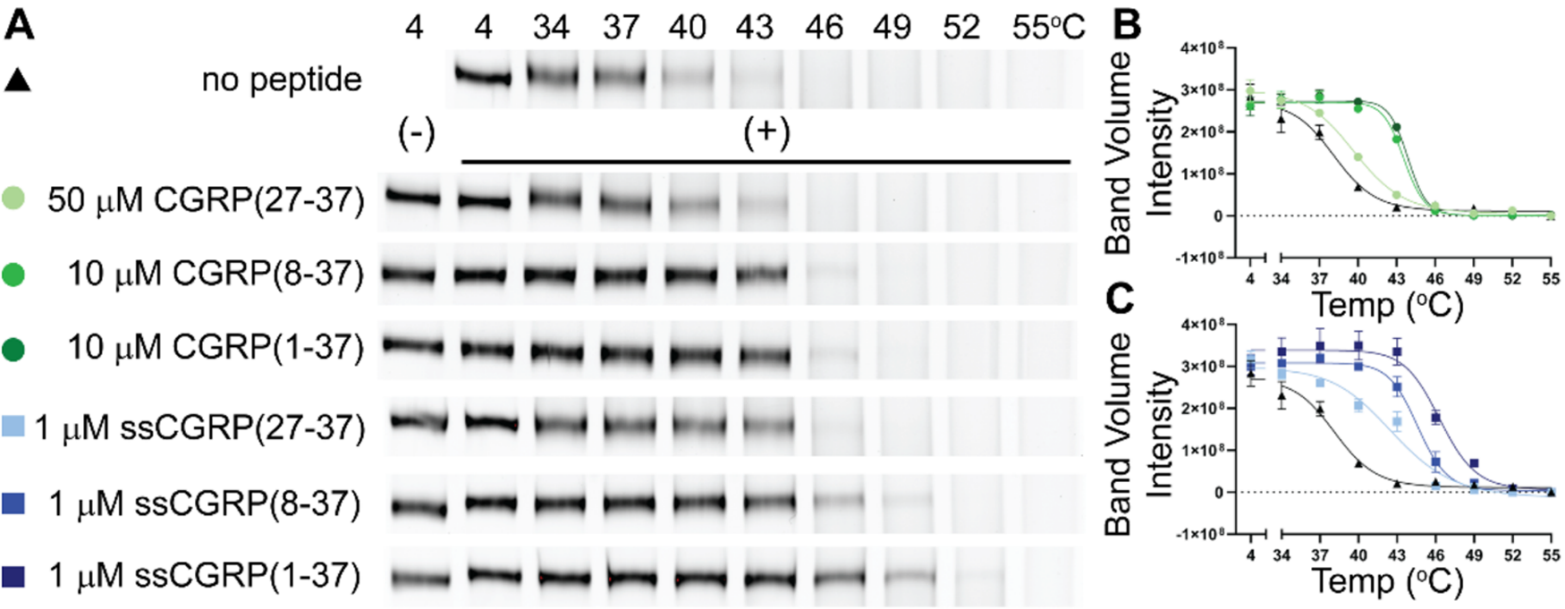
CGRP and ssCGRP effects on heterodimer thermostability. (A) Heterodimer thermostability native PAGE assay with membranes expressing MBP-CLR-eGFP and MBP-RAMP1 from HEK293S GnT1^–^ cells. The detergent-solubilized membranes were incubated at the indicated temperatures in the absence or presence of the indicated peptides followed by native PAGE analysis. Gels were imaged using eGFP in-gel fluorescence. Gels are representative of three independent experiments and show the CGRPR heterodimer band. (B and C) Densitometry analysis showing the melting curves with CGRP (B) or ssCGRP (C). The no peptide control was plotted on both graphs for reference. Plots combined *n* = 3 independent replicates with mean ± SEM.

To further explore the ability of the antagonist and agonist peptides to engage the two receptor domains, we analyzed their effects on stability of the LMNG/CHS detergent-solubilized CGRPR using a previously described native PAGE thermostability assay^22,29,33^ in which the uncoupled state of the receptor is analyzed. The ligand-free CGRPR had an apparent melting temperature of 38.2 °C, which was further stabilized to 39.8 °C by CGRP(27-37) occupancy, and to 43.6 °C by CGRP(8-37) or 43.9 °C (1-37) occupancy (Figure 5A,B; Table S4). Each of the ssCGRP peptides progressively further raised the CGRPR melting temperature to 42.7, 44.6, and 46.4 °C for the (27-37), (8-37), and (1-37) fragments, respectively (Figure 5A,C; Table S4). These results are consistent with the (8-37) and (1-37) peptides engaging the receptor TMD, thereby leading to improved thermostability over that observed with the (27-37) fragment that only engages the ECD.

### Characterization of the (1-37) Agonists

ssCGRP(1-37) exhibited a similar signaling potency to wt CGRP(1-37) at CLR:RAMP1 while having ∼100-fold stronger signaling potencies at CLR:RAMP2/3 in an end point equilibrium cAMP accumulation assay.^26^ These data seemed to suggest that ssCGRP was a nonselective agonist for the three CLR:RAMP complexes. However, ssCGRP was long-acting at the CGRPR in an agonist wash-out cAMP accumulation assay.^26^ Here, we characterized the binding and signaling properties of the two full-length CGRP agonists using the new BRET assays to define their receptor affinities, signaling durations, and receptor selectivity profiles more rigorously.

**Figure 6:**
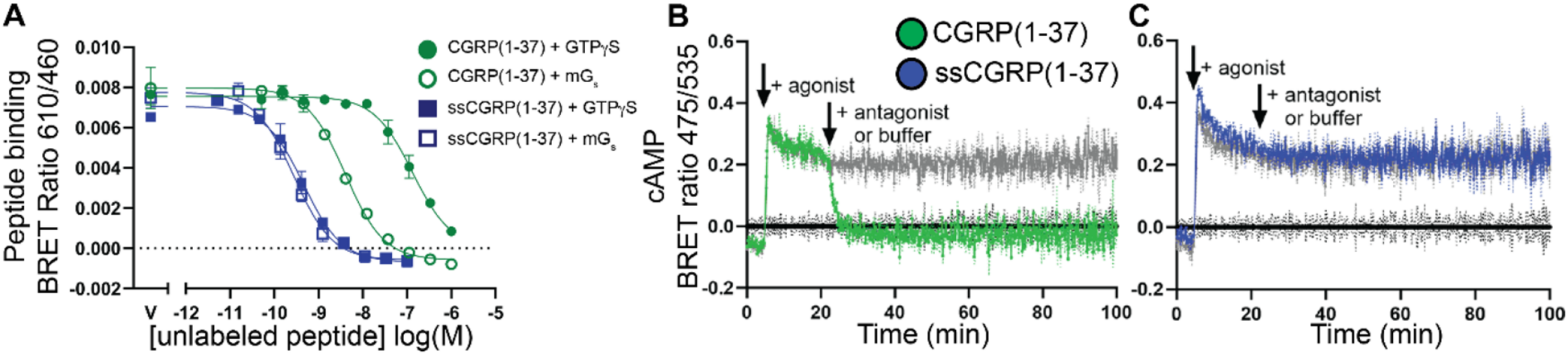
Equilibrium binding and signaling kinetics of CGRP and ssCGRP (1-37) peptides. (A) nanoBRET competition equilibrium in COS-7 membranes competing against 30 nM AM2/IMD(8-47)-TAMRA probe in the G protein uncoupled state (50 μM GTPγS) or 3 nM probe in the G protein coupled state (30 μM mG_s_). (B, C) cAMP signaling kinetics in COS-7 cells expressing CGRPR. Cells were stimulated with 100 nM CGRP(1-37) (B) or ssCGRP(1-37) (C) followed by 10 μM addition of ssCGRP(8-37) antagonist or buffer addition (gray).

Equilibrium nanoBRET competition peptide binding experiments were used to characterize binding of the wt and ssCGRP(1-37) agonists to both the uncoupled (with GTPγS) and G protein-coupled (with miniGs) states of the receptor, again using the AM2/IMD-TAMRA agonist probe. CGRP(1-37) bound the uncoupled state with a *K_i_* of 74 nM and the coupled state with a ∼25-fold stronger *K_i_* of 3 nM (Figure 6A; Table S5). As expected, ssCGRP(1-37) bound the uncoupled state much more strongly than wt CGRP(1-37), having a *K_i_* of 0.25 nM. Unexpectedly, ssCGRP(1-37) did not display further enhanced affinity for the coupled state (*K_i_* = 0.2 nM) (Figure 6A; Table S5). Thus, ssCGRP(1-37) appeared to lose sensitivity to G protein in terms of the classic allosteric G protein effect on agonist affinity.

Signaling by the agonists was characterized in the functional cAMP biosensor assay with the CGRPR expressed in COS-7 cells using a previously described format in which an agonist stimulation period is followed by challenge with an excess of a high-affinity antagonist,^29,34^ in this case ssCGRP(8-37). The rate of cAMP decay after antagonist addition provides a measure of signal duration and may be a proxy for the agonist off-rate. In agreement with our prior studies,^29,34^ CGRP(1-37) was a short-acting agonist with a cAMP decay rate of 0.3 min^–1^ and half-life of 2.4 min (Figure 6B; Table S5). In striking contrast, ssCGRP(1-37) was a very long-acting, sustained signaling agonist with no detectable cAMP decay observed even after 80 min of antagonist challenge (Figure 6C; Table S5).

To define their receptor selectivity profiles, the agonists were also examined at CLR-RAMP2/3 by challenge with the previously described high-affinity ssAM(22-52) antagonist^26^ (Figure S4). ssCGRP(1-37) was short-acting at the RAMP2 complex with a half-life of 3.2 min, and longer acting at the RAMP3 complex with a half-life of 17 min, albeit not as long-acting as at the CGRPR (Table S5). These results suggested that ssCGRP(1-37) is kinetically selective for the CGRPR.

### Reaction Network Modeling of Equilibrium Two-Domain CGRPR Binding

**Figure 7:**
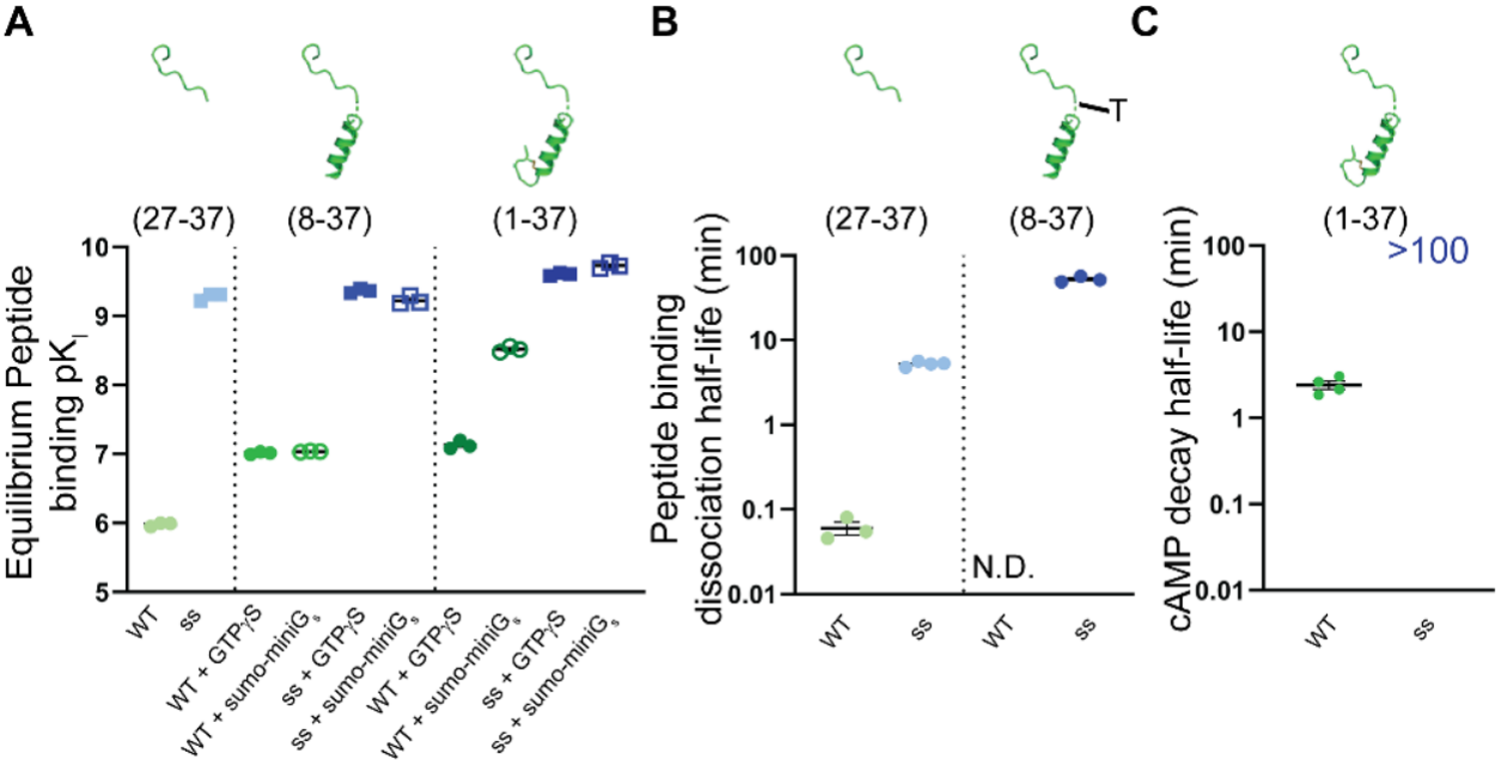
Summary scatter plots of peptide affinity and half-life values. (A) Scatter plot summarizing the p*K*_i_ values from nanoBRET competition equilibrium assays for each length of CGRP and ssCGRP peptide. (B) Scatter plot summarizing the peptide binding half-life values from nanoBRET competition Motulsky-Mahan kinetics for CGRP and ssCGRP for (27-37) lengths and the slow half-life value of ssCGRP(8-37)-TAMRA. The “T” is for the TAMRA label in the peptide depiction for the (8--37) peptide. (C) Scatter plot summarizing the cAMP signaling decay half-life values for CGRP and ssCGRP (1-37). ssCGRP(1-37) was not able to be determined experimentally. All plots show the mean ± SEM of at least three individual replicates.

The equilibrium binding affinities, dissociation half-lives, and cAMP decay half-lives observed for the three different lengths of the wt and ss CGRP peptides at the CGRPR are summarized in Figure 7. The behavior of the wt CGRP fragments was consistent with the classical two-domain model for class B GPCRs; the two longer fragments had stronger equilibrium affinities and slower dissociation rates than the ECD-binding (27-37) fragment, consistent with their engagement of both sites. In addition, the wt agonist displayed a higher affinity for the G protein-coupled state of the receptor as is commonly observed for GPCRs. In contrast, the equilibrium binding affinities of the three ss fragments were similar to each other and binding of the ss agonist was insensitive to the G protein allosteric effect.

**Figure 8:**
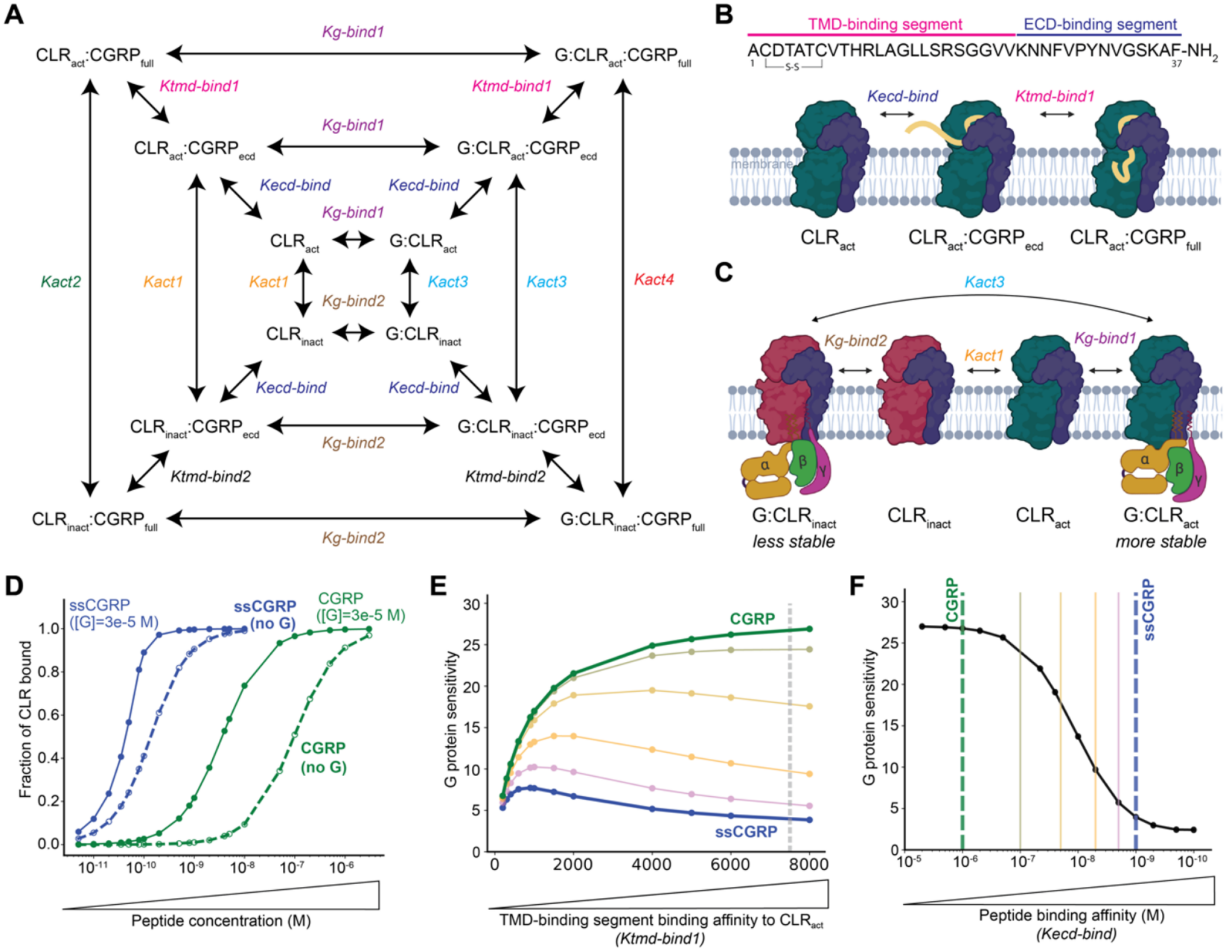
The reaction network model and mechanistic analysis of CLR activation and peptide binding in the presence and absence of G proteins. (A) The reaction network illustrates peptide and G protein binding steps for CLR in both active and inactive states and the corresponding equilibrium constant (*K*) for each step is shown (see “the reaction network” section in Methods for a detailed explanation). (B) Top: CGRP primary sequence. The sequences of two binding segments are color coded. Bottom: Depiction of two-stage binding mechanism of CGRP peptide to *CLR*_*act*_. (C) Depiction of conformational change and G protein binding mechanism of active (CLR_act_) and inactive (CLR_inact_) CLR. (D) Fraction of CLR occupied by both CGRP (green) and ssCGRP (blue) in the presence (solid line/filled circles) and absence (dashed line/open circles) of G proteins. This accounts for all four fully CGRP-bound species (CLR_act_:CGRP_full_, CLR_inact_:CGRP_full_,, G:CLR_act_:CGRP_full_, and G:CLR_inact_:CGRP_full_). (E) G protein sensitivity is plotted against the binding affinity of the TMD-binding segment of the peptide to CLR_act_ (*K*_tmd-bind1_). This is shown for CGRP (green), ssCGRP (blue) and intermediate points with gradual change between CGRP and ssCGRP. All G protein sensitivity plots are obtained from a ratio of half-maximum values in the absence and presence of G proteins, which is calculated by fitting the binding curves (e.g., panel D) to a sigmoid function. The *K*_*tmd-bind1*_ value used to obtain plot D is shown with a gray dashed line. (F) G protein sensitivity vs binding affinity of the ECD-binding segment of peptide (*K*_ecd-bind_) is shown. Specific *K*_ecd-bind_ values for CGRP and ssCGRP are marked by vertical green and blue dashed lines, respectively. Intermediate values are shown with solid vertical lines, transitioning from blue to green to indicate the gradual change. Images created with Biorender.com.

To further probe the CGRPR two-domain model, we constructed an equilibrium reaction network model that describes CLR agonist peptide binding, activation, and G protein binding, to compare the binding profiles of the wt and ss peptide agonists to CLR both in the presence and absence of G protein. In this reaction network model (Figure 8A), binding of the peptide occurs in two stages: an initial ECD-binding step, followed by TMD binding (Figure 8B) and CLR is either in the “active” state (CLR_act_) or the “inactive” state (CLR_inact_*)*. In the absence of peptide and G protein, only a small fraction of CLR is active, but the active state is stabilized by both the presence of G protein and binding of the TMD-binding segment of the peptide. The equilibrium constant of the initial ECD-binding step (*K*_ecd-bind_) is assumed to be independent of both G protein binding and CLR activation. As shown in Figure 8C, the trimeric G protein complex (denoted by “G”) can bind to both active (dark green) and inactive (red) CLR. The enhanced stability of the G:CLR_act_ complex is determined by a numerical constant, β (see eq 3 in Methods), which sets both the ratios *K*_act1_/*K*_act3_ and *K*_g-bind1_/*K*_g-bind2_. Unless stated otherwise, we use β = 1 × 10^–5^, indicating that G protein binding is 100,000 times more stable to the activated form of CLR, although we note that the results are qualitatively consistent across a wide range of β values (Figure S6E). In this model, CLR activation acts as a simple allosteric connection between peptide binding and G protein binding.

After all equilibrium constants are specified, the concentrations of each individual species in the model can be solved, under the restraints of the total concentrations for CLR, G protein and peptide (see “The reaction network” and “Solving the reaction network” sections in Methods for more detailed explanation). Figure 8D shows the total fraction of CLR that is bound as a function of peptide concentration which can be directly compared to experimental results in Figure 6A. We find that the model reproduces the experimentally observed behavior of wt CGRP. The presence of G protein significantly enhanced the binding affinity of CGRP. Interestingly, the model predicted that the binding affinity of ssCGRP should also be sensitive to the G protein allosteric effect, albeit to a lesser extent than that of CGRP. This contrasts with the experimental results, which show no detectable G protein sensitivity for ssCGRP. These results are quantified using *K*_d_ values, calculated by fitting the binding curves to a sigmoid function and as a result, *K*_d_ values of 96, 3.6, 0.14, and 0.040 nM are obtained for free CGRP, CGRP with G protein bound, free ssCGRP, and ssCGRP with G protein bound. This shows us a 27- and 3.6-fold change upon addition of G protein (hereafter referred to as “G protein sensitivity”) for CGRP and ssCGRP binding, respectively. Thus, this simple two-domain binding model could explain part of the observed differences in G protein sensitivity between CGRP and ssCGRP, however it does not capture the complete loss of G protein sensitivity exhibited by ssCGRP experimentally.

The model behavior was robust to changes in different model parameters. CGRP showed a consistent increase in G protein sensitivity with increasing TMD binding affinity (*K*_tmd-bind1_), while ssCGRP exhibited some reduced sensitivity with a rise-and-fall pattern (Figure 8E). We examined intermediate values of the ECD binding affinity (*K*_ecd-bind_), and found that G protein sensitivity follows a sigmoidal pattern that falls with increasing peptide binding affinity (Figure 8F). To further provide insight into the relationship between ECD affinity, TMD affinity, and G protein sensitivity, the intermediate values of *K*_ecd-bind_ from Figure 8F were examined over different TMD binding affinities in Figure 8E. As the ECD binding affinity increases, there is a gradual decrease in G protein sensitivity at higher TMD binding affinities. The intermediate ECD binding affinities also exhibit the distinctive rise-and-fall pattern, where a maximum G protein sensitivity value is reached for intermediate values of the TMD binding affinity.

To examine the mechanism more deeply, we plot the fraction of CLR that is in the active state as a function of peptide concentration (Figure S6A). In the absence of G protein, most of CLR remains inactive, even at high concentrations of either peptide. In the presence of G protein, CLR largely becomes activated in a manner that tracks the CLR peptide binding curves in Figure 8D. This shows that while G proteins are not necessary for peptide binding, they are crucial for effective CLR activation, and it underlines the role of G proteins in stabilizing the active conformation of CLR for peptide binding. The reduction in G protein sensitivity of ssCGRP compared to CGRP was robust to other model parameters as well. The G protein sensitivity was consistently higher for CGRP compared to ssCGRP as a function of G protein binding affinity (*K*_g-bind1_) (Figure S6B).

This difference grew with *K*_g-bind1_ and shrank as the G protein binding affinity decreased below 10^7^ M. Additionally, as baseline CLR activation (*K*_act1_) increased, CGRP showed a significant fold change, while ssCGRP changed to a much lesser extent (Figure S6C). Varying the α value, which represents the factor by which TMD binding affinity is increased upon CLR activation, shows a smooth increase in G protein sensitivity for CGRP, while ssCGRP again shows a substantially weaker response (Figure S6D). Examining G protein sensitivity as a function of the β value – which reflects how activation enhances G protein binding – shows a strong increase in G protein sensitivity for CGRP with decreasing β, which is much reduced for ssCGRP (Figure S6E).

We also examined the behavior of the model under the conditions of varying CLR concentration. Figure S7 shows the fraction of bound CLR as a function of peptide concentration for CLR concentrations ranging from 2 nM to 10 pM. Recall that 80 pM was used in Figure 8, which is CLR concentration in our experimental conditions. We find for CGRP, the G protein sensitivity changes from ∼27 (at low [CLR]) to ∼21 (at high [CLR]), while for ssCGRP, the G protein sensitivity decreases dramatically with CLR concentration, with values of 12.04, 2.98, 1.29, and 1.12 for [CLR] = 10 pM, 100 pM, 1 nM and 2 nM, respectively.

In summary, our model consistently shows higher G protein sensitivity for CGRP compared to ssCGRP. The conditions for which CGRP G protein sensitivity is diminished correspond either to the decoupling of the allosteric mechanism (α→1, β→1, *K*_act1_→0), or disruption of G protein binding (*K*_g-bind1_→0) or TMD binding (*K*_tmd-bind1_→0) entirely. However, the ssCGRP G protein sensitivity in the model is higher than that observed experimentally, except for at concentrations of CLR (e.g., >1 nM) that are known to be higher than our experimental conditions.

## Discussion

Cryo-EM structural studies conducted over the last several years confirmed that the two-domain peptide binding mechanism applies to all 15 human class B GPCRs, at least with respect to how the peptide agonists engage the receptor in the active-state Gs-bound complexes.^43,44^ Less is known about the steps leading to formation of the active state complexes. In addition, the quantitative aspects of the mechanism are poorly defined for many of the receptors. Here, we quantitatively characterized the two-domain mechanism for the CGRPR, which is a proven drug target for migraine headache and has potential as a therapeutic target for several other indications. The mechanism was experimentally explored in receptor binding and signaling assays for its endogenous ligand CGRP and our engineered ultrahigh affinity variant ssCGRP. We also presented a mathematical reaction network model to investigate the consequences of the two-domain binding mechanism. Together, these experimental and theoretical studies provide unique insights into the CGRPR ligand binding and receptor activation mechanism and better define the equilibrium and kinetic properties of the ssCGRP agonist and truncated antagonist variants.

Prior studies characterized the equilibrium binding affinities of the wt and ssCGRP(27-37) ECD-binding antagonist fragments and their antagonism of cAMP signaling,^25,26,36,38^ but their kinetics of binding had not been reported. Using the new nanoBRET receptor binding assay, we obtained equilibrium binding affinities that were in good agreement with the prior studies. The kinetic competition nanoBRET binding experiments revealed that ssCGRP(27-37) had a ∼16-fold faster on rate and ∼90-fold slower off rate than wt CGRP(27-37). This resulted in a receptor residence time of ∼8 min for the ss variant as compared to only ∼5 s for wt. These rate changes may result from the ss substitutions stabilizing the β-turn structure present in the receptor-bound conformation (Figure 1C). These nanoBRET assays (Figure 2 and Table S2) indicated that ssCGRP(27-37) is a “fast on/slow off” CGRPR antagonist with a high affinity encroaching into the picomolar range. In contrast, wt (27-37) is a “slow on/fast off” antagonist with moderate μM affinity.

Extending the peptides longer in the (8-37) antagonist backbone provides the potential for two-site binding. Nonetheless, whether the (8-37) antagonists engage the TMD was an open question, particularly because the G protein-free CGRPR cryo-EM structure showed the CGRP(1-37) agonist to be predominantly single-site ECD-engaged.^23^ The affinity of the CGRP(8-37) antagonist was previously characterized in receptor binding assays and in functional antagonism of cAMP signaling assays. Several studies reported single digit nM affinity,^39,45−48^ but higher affinities in the pM range^38,49−51^ and weaker affinities as low as 800 nM^52−54^ have also been reported. In our nanoBRET equilibrium competition binding assays, CGRP(8-37) had an affinity of ∼95 nM irrespective of the receptor being in the uncoupled or G protein-coupled states. The ∼10-fold higher affinity of (8-37) than (27-37) was consistent with it making additional contacts to the TMD, and this was further supported by the antagonism of AM(13-36) signaling and thermostability assays (Figures 4 and 5). In contrast, the equilibrium affinity of ssCGRP(8-37) (0.43 to 0.6 nM) was about the same as that of ssCGRP(27-37). Despite this, ssCGRP(8-37) also appeared to engage the TMD in the Figures 4 and 5 experiments. One possible explanation for this apparent discrepancy is that enthalpy gain from new TMD contacts was offset by an increased loss of binding entropy. Overall, these data were consistent with both (8-37) antagonists engaging the TMD, however, the extent of these interactions is uncertain. We cannot exclude the possibility that the behavior of the (8-37) fragments in these assays reflected limited contact with the top portion of the TMD and/or effects of the N-terminal extension on ECD-binding.

The affinity and kinetics of binding of the ssCGRP(8-37) antagonist were further examined in the nanoBRET binding assay using a TAMRA-labeled version (Figure 3 and Table S3). These data revealed a high affinity *K*_d_ of 390 pM and two-phase dissociation kinetics consistent with a two-site binding mechanism. The fast dissociation component may reflect complexes in which the antagonist was single-site ECD engaged. Indeed, this component yielded a residence time (19 min) similar to that of ssCGRP(27-37). The slow dissociation component may reflect two-site engagement, and for these complexes the residence time was 76 min. This value is likely more accurate than the 12 h residence time that we estimated in our prior study using the hemiequilibrium operational model data analysis approach.^26^ Despite this revision, ssCGRP(8-37) can still be considered a very slow off-rate antagonist with a residence time measured in hours.

In the nanoBRET equilibrium competition binding experiments, the two full-length (1-37) agonist peptides exhibited affinities for the uncoupled state of the receptor that were slightly stronger than the (8-37) antagonists (Figure 7 and Table S5). The thermostability assays were consistent with the agonists engaging the TMD even in the uncoupled state (Figure 5). For wt CGRP(1-37), the G protein allosteric effect increased its affinity ∼25-fold. This is considerably higher than the ∼6-fold *K*_d_ change observed for the AM2/IMD-TAMRA agonist at the CGRPR (CLR:RAMP1) in this study (Table S1). In addition, our prior study of the binding of AM2/IMD-TAMRA and AM-TAMRA to the AM_2_R (CLR:RAMP3) indicated that these agonists had only ∼2-fold and ∼3-fold increased *K*_d_, respectively, for the coupled state over the uncoupled state.^29^ These data suggest that wt CGRP is a higher efficacy agonist than the two AM peptides. This is further supported by our prior study that showed that the CGRP-occupied CGRPR had a higher apparent affinity for mGs (∼200 nM) than the AM- or AM2/IMD-occupied CGRPR or AM_2_R (apparent mGs affinities ∼5 to 10 μM).^22^ Notably, the G protein allosteric effect was largely absent for ssCGRP(1-37), which had similar strong picomolar affinities for the two receptor states.

Complementary to our experimental findings, the reaction network model allowed a quantitative characterization of the two-domain mechanism for the CGRPR. With this model, we showed that a simple allosteric mechanism – in which both TMD binding and G protein binding can influence a binary conformational switch – was sufficient to model the experimental behavior of the wt CGRP agonist. In contrast, although the model exhibited some differences in G protein sensitivity, the model did not fully capture the experimental behavior of the ssCGRP agonist. According to the model, ssCGRP should have retained some sensitivity to the G protein allosteric effect, but this was not observed in our experiments. One possible explanation is that ssCGRP G protein sensitivity was not observed because we did not reach equilibrium in the binding assays. While we cannot exclude this possibility, we think it is unlikely because the binding reactions were incubated for 8 h. An alternative explanation is that ssCGRP does not fit the assumptions of our reaction network model. For instance, the unique ECD binding domain of ssCGRP could induce an allosteric change in CLR that either alters peptide binding affinity in the TMD region, or affects CLR activation. Extending this model to describe other possible CLR conformations, such as those compatible with β-arrestin recruitment, could enable a more quantitative description of differential agonism. The model presented here is also limited to studies in equilibrium. Binding and unbinding rates, together with rates of CLR conformational change, would be needed to study nonequilibrium effects on signaling duration that could arise from time-varying peptide concentrations.

Notably, there are other examples in the literature of apparent loss of G protein sensitivity with increased affinity of class B GPCR agonists. Andreassen et al.^55^ showed differences in G protein sensitivity for two peptide agonists of the calcitonin (CT) receptor (CTR). Salmon calcitonin (sCT) binds to the human CTR with high affinity and long residence time and is G protein-insensitive, whereas human CT shows a clear preference for the G protein-coupled state and has a shorter residence time. Similar to ssCGRP, the C-terminal segment of sCT has increased affinity for the receptor ECD, being ∼50-fold higher affinity than hCT.^56^ However, the sCT substitutions responsible for its slower off-rate are in the TMD-binding segment.^57^ Another example was observed by Okazaki et al.^58^ in variants of parathyroid hormone (PTH) binding to the PTH1R. In this case, the wildtype PTH(1-34) and modified M-PTH(1-34) variant differed only by a series of mutations in the TMD-binding N-terminal half of the peptide. M-PTH(1-34) displayed ∼24-fold enhanced affinity for the uncoupled state of PTH1R (R^0^ in their terminology), while its affinity for the coupled state was largely unchanged. M-PTH(1-34) also displayed prolonged cAMP signaling. Clearly, increasing ECD binding affinity of a class B GPCR agonist is not the only route to apparent insensitivity to the G protein allosteric effect and/or long-duration signaling.

We examined the signaling durations of the wt and ss agonists in the CAMYEL cAMP biosensor assay as measured by the cAMP decay rate after antagonist challenge (Figure 6B,C). These assays showed that wt CGRP(1-37) is a short-acting agonist at the CGRPR with a *t*_1/2_ of 2.4 min, whereas ssCGRP(1-37) is a very long-acting, sustained signaling agonist. We were not able to measure the decay rate, but the ss agonist clearly had a signal duration measured in hours under our experimental conditions. These experiments also advanced our understanding of the selectivity profile of ssCGRP(1-37). While our previous study indicated that it was nonselective for the three CLR-RAMP complexes in an end point equilibrium cAMP accumulation assay,^26^ the biosensor signaling duration assays here showed that it is kinetically selective for the CGRPR. The cAMP signal decay after antagonist challenge may be a proxy for the agonist dissociation rate,^29^ but it is unclear if this is the case for ssCGRP because we did not directly measure its unbinding kinetics. This issue is further complicated by the fact that the CGRPR can undergo agonist induced internalization and even wt CGRP can elicit cAMP signaling from internalized receptor.^59,60^ Future work is needed to determine if internalized receptor plays a role in the long-duration signaling of ssCGRP and how this is affected by agonist residence time.

Our analysis of the ssCGRP agonist and its truncated antagonist forms advance our understanding of their potential as long-acting therapeutics. It is widely accepted that long residence time is a desirable property of drugs.^61,62^ This is particularly true for antagonists, but there can also be value in long residence time GPCR agonists.^63,64^ Like many peptides, CGRP has a short plasma half-life of ∼20 min,^65^ which limits its use as a therapeutic. The ssCGRP(8-37) antagonist has a CGRPR residence-time longer than the plasma half-life and the ssCGRP agonist can signal for longer than the plasma half-life. These features could make them valuable as long-acting therapeutics suitable for less frequent dosing. Nonetheless, future work will be needed to assess their suitability as drugs in animal models, particularly for the ssCGRP(1-37) agonist because the cellular consequences of sustained CGRPR activation remain unclear.

## Conclusions

This study provided mechanistic insights into the “two-domain” peptide binding mechanism of the human CGRP receptor for the wild-type αCGRP ligand and an engineered ultrahigh affinity variant “ssCGRP”. The receptor binding and signaling properties of the three different peptide lengths examined, the truncated (27-37) and (8-37) antagonists, and the full-length (1-37) agonist, supported a mechanism in which the wild-type agonist first binds the ECD followed by binding and activation of the TMD coincident with G protein binding. Further support for this was provided by a reaction network mathematical model that quantitatively characterized the allosteric communication between peptide and G protein binding to the receptor TMD. Compared to wild-type CGRP, the ss variant exhibited dramatically increased receptor binding affinity, a longer receptor residence time, and prolonged cAMP signaling duration. ssCGRP also exhibited two-domain binding, but it appeared to be insensitive to the allosteric G protein effect on binding affinity. Overall, this study advances our quantitative understanding of the CGRPR two-domain peptide binding mechanism and will facilitate future drug development efforts.

## Data Availability

Raw data will be made available upon reasonable request to the corresponding authors.

## Supporting Information Available

Characterizing the TAMRA labeled probes, cAMP signaling duration at CLR-RAMP2/3, melting temperatures from thermostability, and additional mathematical modeling plots (PDF)
