# Supplementary material for: Characterization of the Two-Domain Peptide Binding Mechanism of the Human CGRP Receptor for CGRP and the Ultrahigh Affinity ssCGRP Variant
Source: Biochemistry. 2025 Apr 2;64(8):1770–87. doi: 10.1021/acs.biochem.4c00812 (PMC12004451; doi:10.1021/acs.biochem.4c00812)
Supplement: Supplementary file 1 — bi4c00812_si_001.pdf [file bi4c00812_si_001.pdf]

Supporting Information for

Characterization of the two-domain peptide binding mechanism of the human CGRP receptor for CGRP and the ultra-high affinity ssCGRP variant

Katie M. Babin[1], Ceren Kilinc[2], Sandra E. Gostynska[1], Alex Dickson[2,3*], and Augen A. Pioszak[1*]

[1]Department of Biochemistry and Physiology, University of Oklahoma Health Sciences Center, Oklahoma City, OK 73104, USA.

[2]Department of Biochemistry and Molecular Biology, Michigan State University, East Lansing, MI 48824, USA.

[3]Department of Computational Mathematics, Science and Engineering, Michigan State University, East Lansing, MI 48824, USA.

*Correspondence: alexrd@msu.edu or augen-pioszak@ouhsc.edu

This file includes:

Figures S1-S7

Tables S1-S5

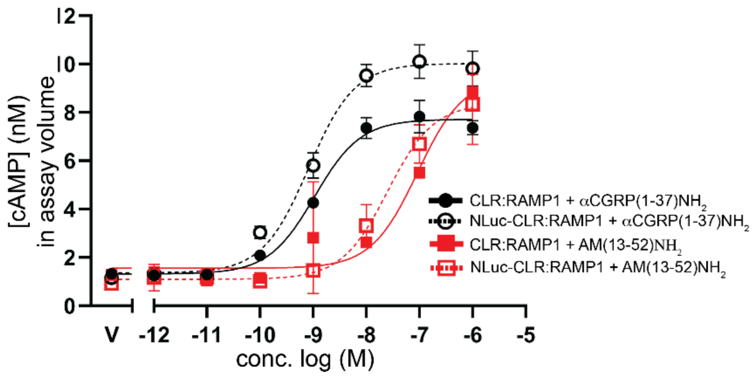

Supporting Information Figure 1: cAMP accumulation assay in COS-7 cells comparing Nluc-CLR co-expressed with RAMP1 to wild-type CLR co-expressed with RAMP1 for αCGRP(1-37) and AM(13-52) agonists.

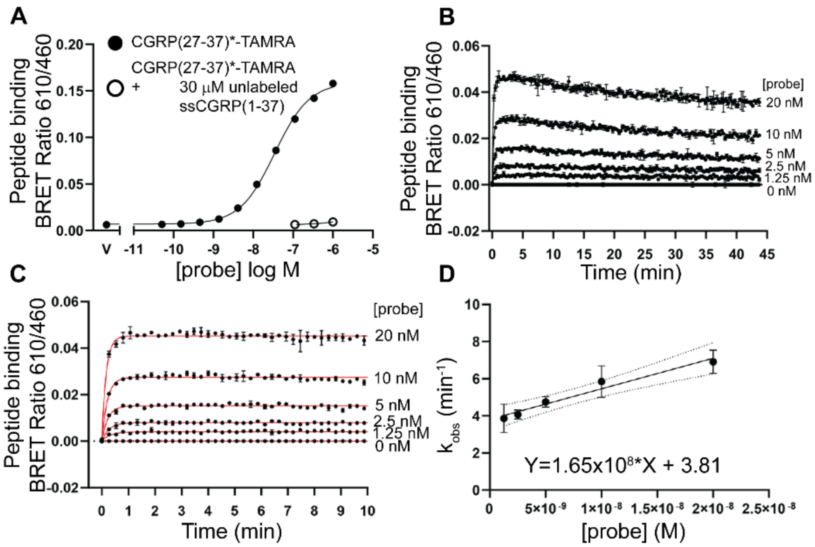

Supporting Information Figure 2: nanoBRET equilibrium and kinetic characterization of CGRP(27-37)*-TAMRA probe. A) Saturation equilibrium binding of CGRP(27-37)*-TAMRA probe in COS-7 membranes with 30 μM ssCGRP(1-37) non-specific binding control. **B)** Association binding kinetics of CGRP(27-37)*-TAMRA in COS-7 membranes with signal decay. **C)** Association kinetics of CGRP(27-37)*-TAMRA probe in COS-7 membranes. Curves were fit using the first ten minutes of data collection. **D)** Linear plot of the association rates from C plotted against the probe concentration. Plot combines all n=3 independent replicates with mean ± SEM.

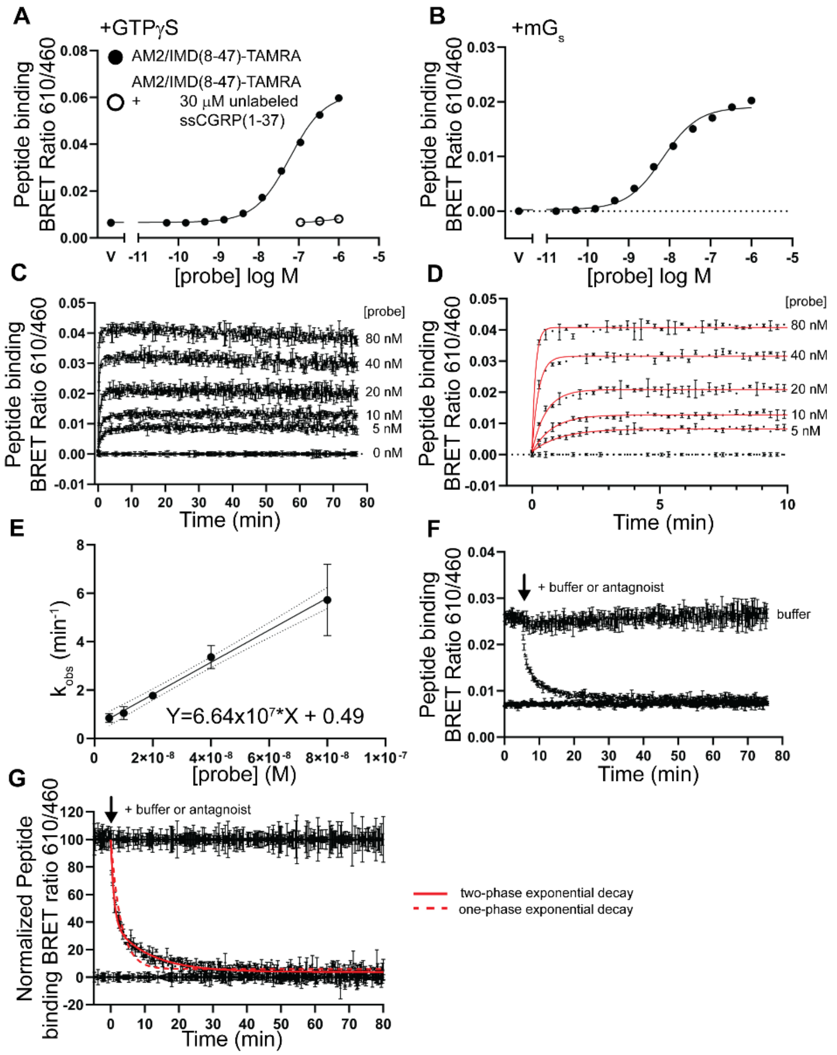

Supporting Information Figure 3: nanoBRET equilibrium and kinetic characterization of AM2/IMD(8-47)-TAMRA probe. A) Saturation equilibrium binding in COS-7 membranes of the G protein uncoupled state (50 μM GTPγS) with a 30 μM ssCGRP(1-37) non-specific binding control. **B)** Saturation equilibrium binding in COS-7 membranes of the G protein coupled state (30 μM mG_s). **C and D)** Association kinetics of AM2/IMD(8-47)-TAMRA probe in COS-7 membranes. Curves were fit using the first ten minutes of data collection (D). **E)** Linear plot of the association rates from D plotted against probe concentration. Plot combines all n=3 independent replicates with mean ± SEM. **F and G)** Dissociation kinetics of 10 nM AM2/IMD(8-47)-TAMRA. Dissociation was initiated by addition of 1 μM ssCGRP(8-37). **F)** Data before membrane only control subtraction and normalization to the buffer addition control. **G)** Normalized data with curve fitting to a one-phase (dashed line) or two-phase (solid line) exponential decay.

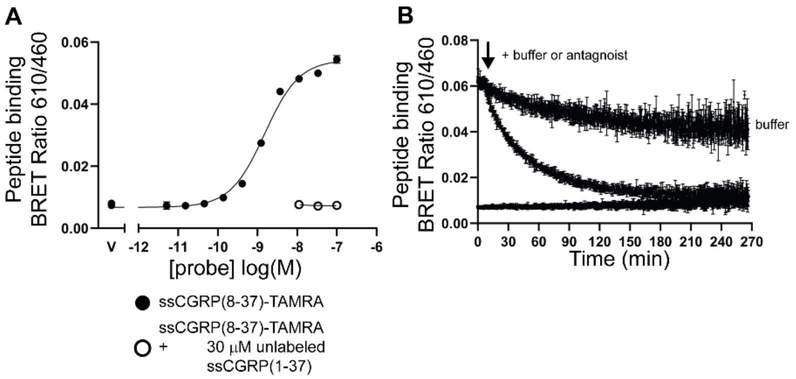

Supporting Information Figure 4: nanoBRET equilibrium and kinetic characterization of ssCGRP(8-37)-TAMRA probe. A) Saturation equilibrium binding of ssCGRP(8-37)-TAMRA probe in COS-7 membranes with 30 μM ssCGRP(1-37) non-specific binding control. **B)** Dissociation binding kinetics of ssCGRP(8-37)-TAMRA before membrane only control subtraction and normalization to the buffer addition control.

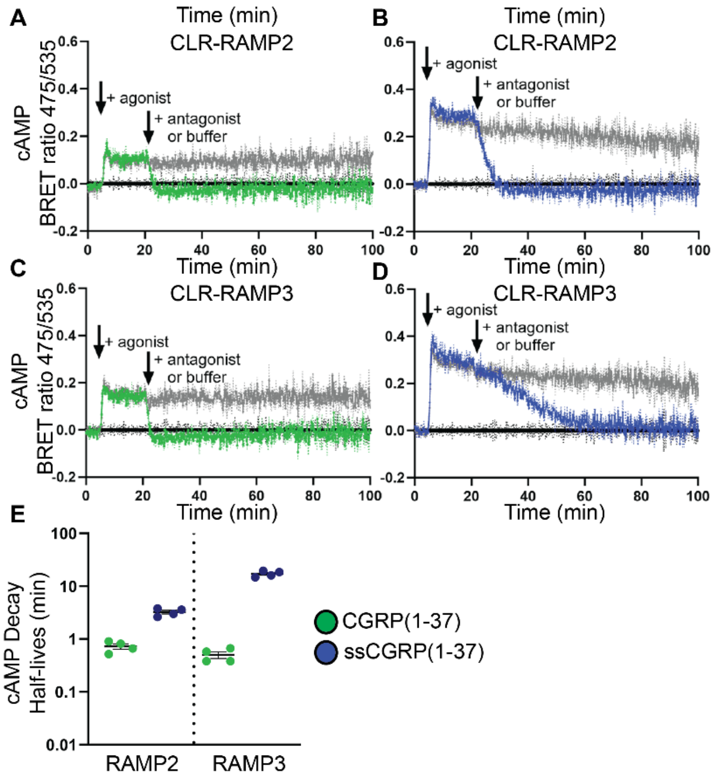

Supporting Information Figure 5: cAMP signaling kinetics of CGRP and ssCGRP (1-37) at CLR-RAMP2 and CLR-RAMP3. A-D) cAMP signaling kinetics in COS-7 cells at the indicated receptor. Cells were stimulated with 100 nM of CGRP or ssCGRP(1-37) followed by addition of 10 µM AM(22-52) S48G/Q50W or buffer (grey). **E)** Scatter plot summarizing cAMP decay half-lives from A-D with mean ± SEM for four independent replicates.

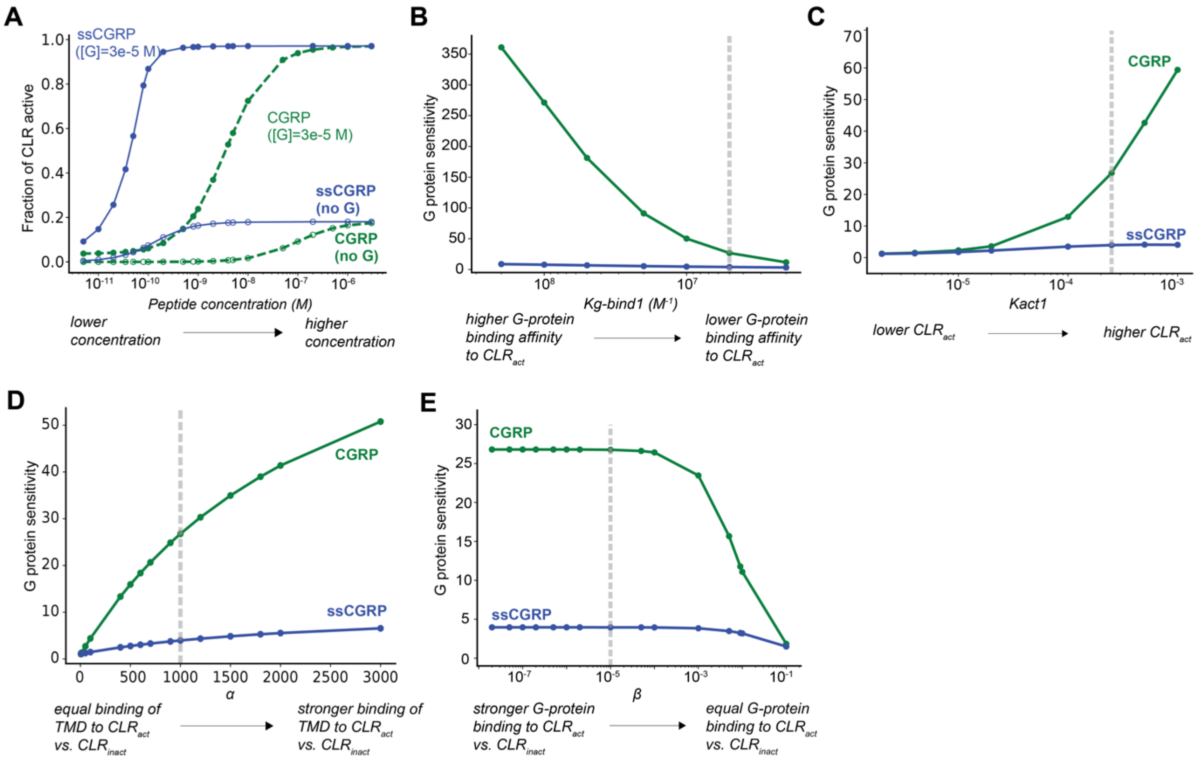

Supporting Information Figure 6: Mechanistic analysis of CLR activation and peptide binding in the presence and absence of G proteins under different parameters of the reaction network model. A) Fraction of activated forms of CLR as a function of peptide concentration, determined for both CGRP (green) and ssCGRP (blue) in the presence (solid line/filled circles) and absence (dashed line/open circles) of G proteins. This accounts for all 6 activated forms of CLR, shown in the top half of **Fig. 8A**. **B)** G protein sensitivity changes as a function of G protein binding affinity to CLR_{act} ($K_{g\text{-}bind1}$) for CGRP (green) and ssCGRP (blue). **C)** G protein sensitivity as a function of CLR_{act} activation baseline (K_{act1}) for CGRP (green) and ssCGRP (blue). **D)** G protein sensitivity as a function of the parameter alpha (α), which governs the factor by which TMD binding is reduced when CLR is inactive (i.e. $\alpha = K_{tmd\text{-}bind1}/K_{tmd\text{-}bind2}$). **E)** G protein sensitivity as a function of the parameter beta (β), which governs the factor by which activation is stronger when G proteins are bound to CLR. Values used to obtain **Fig. 8D** is shown with a gray dashed line.

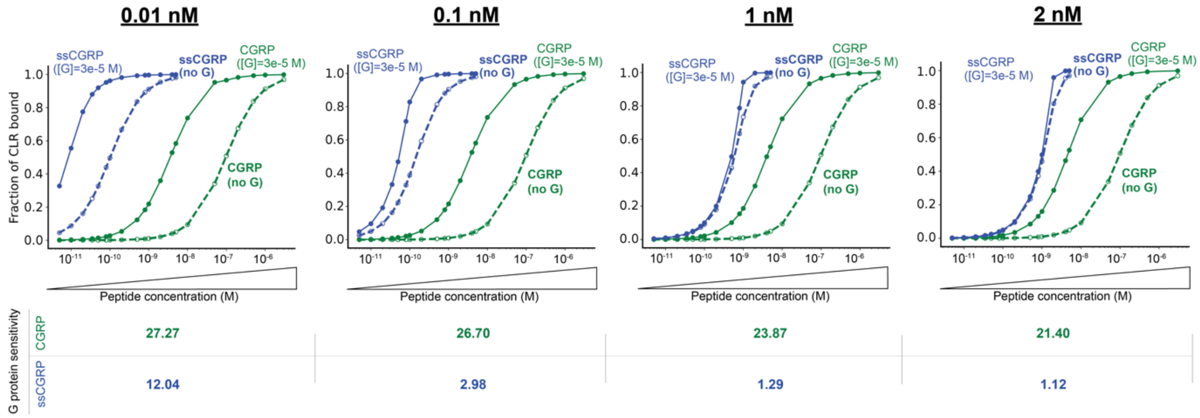

Supporting Information Figure 7: Analysis of G protein sensitivity as a function of CLR concentration. The fraction of CLR that is bound is plotted as a function of peptide concentration for total CLR concentrations ranging from 0.01 nM to 2.0 nM. Solid curves show data in the presence of G protein ([G] = 3e-5 M) and dashed curves show the absence of G protein ([G] = 0 M). The G protein sensitivity, or fold-change upon addition of G protein, is tabulated below, for CGRP and ssCGRP.

Supporting Information Table 1: Summary of CGRP(27-37)*-TAMRA and AM2/IMD(8-47)-TAMRA peptide probes binding at Nluc-CLR-RAMP1 membranes.[*]

		Peptide Binding	
		CGRP(27-37)*-TAMRA	AM2/IMD(8-47)-TAMRA
Eq[†]	K_d + GTPγS (nM ± SEM)	28.72 ± 2.57	40.98 ± 1.67
	K_d + mG_s (nM ± SEM)	NA[‡]	6.51 ± 0.53
association[§]	k_{on} ($M^{-1}min^{-1}$ ± SEM)	$1.65x10^8 \pm 6.31x10^6$	$6.64x10^7 \pm 1.09x10^7$
	k_{off} (min^{-1} ± SEM)	3.81 ± 0.27	0.49 ± 0.13
	K_d calc[**] (nM ± SEM)	22.54 ± 0.81	8.14 ± 2.93
	Residence time[††] (min)	0.27 ± 0.02	2.29 ± 0.49
	$t_{1/2}$[‡‡] (min)	0.18 ± 0.01	1.59 ± 0.34
dissociation[§ §]	k_{off} fast (min^{-1} ± SEM)	NA	1.25 ± 0.08
	k_{off} slow (min^{-1} ± SEM)	NA	0.106 ± 0.008
	Residence time fast (min)	NA	0.81 ± 0.06
	Residence time slow (min)	NA	9.50 ± 0.69
	$t_{1/2}$ fast (min)	NA	0.56 ± 0.04
	$t_{1/2}$ slow (min)	NA	6.58 ± 0.48
	Percent fast	NA	61.44 ± 0.84

[*] Values derived from Fig. S2 and S3 experiments. n=3 for each experiment.
[†] Equilibrium values derived from Fig. S2A, S3A, and S3B
[‡] NA = not applicable
[§] Values derived from nanoBRET binding association experiments Fig. S2C, S2D, S3D, and S3E
[**] K_d calculated as the ratio of k_{off}/k_{on}.
[††] Residence time calculated as the inverse of k_{off}.
[‡‡] Half-life calculated as ln 2 divided by k_{off}.
[§§] Values derived from nanoBRET binding two-phase dissociation experiments Fig. S3G

Supporting Information Table 2: Summary of equilibrium and kinetic values of (27-37) peptides derived from binding assays.*

	Peptide Binding	
	CGRP(27-37)	ssCGRP(27-37)
K_i (nM ± SEM)	1,049 ± 39	0.527 ± 0.039
k_{on} ($M^{-1}min^{-1}$ ± SEM)	$1.17x10^7$ ± $1.98x10^6$	$1.93x10^8$ ± $3.10x10^7$
k_{off} (min^{-1} ± SEM)	12.16 ± 1.91	0.133 ± 0.005
K_d calc† (nM ± SEM)	1,043 ± 32	0.73 ± 0.10
Residence time‡ (min)	0.087 ± 0.015	7.57 ± 0.27
$t_{1/2}$§ (min)	0.06 ± 0.01	5.24 ± 0.18

* Values derived from Fig. 2 experiments. n=3 for each experiment.
† K_d calculated as the ratio of k_{off}/k_{on}.
‡ Residence time calculated as the inverse of k_{off}.
§ Half-life calculated as ln 2 divided by k_{off}.

Supporting Information Table 3: Summary of ssCGRP(8-37)-TAMRA peptide probe binding to Nluc-CLR-RAMP1 membranes.[*]

		Peptide Binding
		ssCGRP(8-37)-TAMRA
Eq[†]	K_d (nM ± SEM)	0.39 ± 0.08
association[‡]	k_{on} ($M^{-1}min^{-1}$ ± SEM)	$2.17x10^8 \pm 2.46x10^7$
	k_{off} (min^{-1} ± SEM)	0.018 ± 0.002
	K_d calc[§] (nM ± SEM)	0.085 ± 0.003
	Residence time[**] (min)	55.33 ± 5.46
	$t_{1/2}$[††] (min)	38.35 ± 3.79
dissociation[‡‡]	k_{off} fast (min^{-1} ± SEM)	0.053 ± 0.005
	k_{off} slow (min^{-1} ± SEM)	0.0133 ± 0.0006
	Residence time fast (min)	19.12 ± 1.9
	Residence time slow (min)	75.69 ± 3.49
	$t_{1/2}$ fast (min)	13.25 ± 1.32
	$t_{1/2}$ slow (min)	52.45 ± 2.42
	Percent fast	25.6 ± 3.5

[*] Values derived from Fig. 3 experiments. n=3 for each experiment.
[†] Equilibrium values derived from Fig. 3B
[‡] Values derived from nanoBRET binding association experiments Fig. 3C and D
[§] K_d calculated as the ratio of k_{off}/k_{on}.
[**] Residence time calculated as the inverse of k_{off}.
[††] Half-life calculated as ln 2 divided by k_{off}.
[‡‡] Values derived from nanoBRET binding two-phase dissociation experiments Fig. 3E

Supporting Information Table 4: Melting temperatures of CLR-RAMP1 complex with various peptides.*

	Melting EC_{50} (°C)
No peptide	38.20 ± 0.22
CGRP(27-37)	39.79 ± 0.19
CGRP(8-37)	43.57 ± 0.12
CGRP(1-37)	43.88 ± 0.14
sCGRP(27-37)	42.67 ± 0.45
ssCGRP(8-37)	44.62 ± 0.14
ssCGRP(1-37)	46.35 ± 0.32

* Values derived from Fig. 5A-C. n=3 for each experiment.

Supporting Information Table 5: Summary of equilibrium and kinetic values of (1-37) peptides derived from binding and signaling assays.*

		Peptide Binding		cAMP signaling†	
		CGRP(1-37)	ssCGRP(1-37)	CGRP(1-37)	ssCGRP(1-37)
CLR-RAMP1	K_i + GTPγS (nM ± SEM)	74.4 ± 5.8	0.248 ± 0.008	NA‡	NA
	K_i + mG_s (nM ± SEM)	3.05 ± 0.17	0.19 ± 0.01	NA	NA
CLR-RAMP1	Observed decay rate (min^{-1} ± SEM)	NA	NA	0.30 ± 0.03	NM§
	Time constant τ** (min)	NA	NA	3.5 ± 0.4	NM
	$t_{1/2}$†† (min)	NA	NA	2.4 ± 0.3	NM
CLR-RAMP2	Observed decay rate (min^{-1} ± SEM)	NA	NA	1.0 ± 0.1	0.22 ± 0.02
	Time constant τ (min)	NA	NA	1.0 ± 0.1	4.7 ± 0.4
	$t_{1/2}$ (min)	NA	NA	0.72 ± 0.09	3.2 ± 0.3
CLR-RAMP3	Observed decay rate (min^{-1} ± SEM)	NA	NA	1.5 ± 0.2	0.041 ± 0.003
	Time constant τ (min)	NA	NA	0.7 ± 0.1	24.9 ± 1.6
	$t_{1/2}$ (min)	NA	NA	0.50 ± 0.07	17.2 ± 1.1

* Values derived from Fig. 6 and S5 experiments. n=3 for peptide binding and n=4 for cAMP signaling experiments.
† Values derived from CAMYEL cAMP biosensor assay with agonist followed by antagonist challenge assays
‡ NA = not applicable
§ NM = not measurable
** Time constant τ calculated as the inverse of observed decay rate.
†† Half-life calculated as ln 2 divided by observed decay rate.
